# An early-onset specific polygenic risk score optimizes age-based risk estimate and stratification of prostate cancer: population-based cohort study

**DOI:** 10.1186/s12967-024-05190-y

**Published:** 2024-04-17

**Authors:** Yifei Cheng, Lang Wu, Junyi Xin, Shuai Ben, Silu Chen, Huiqin Li, Lingyan Zhao, Meilin Wang, Gong Cheng, Mulong Du

**Affiliations:** 1 Jiangsu Key Laboratory of Cancer Biomarkers, Prevention and Treatment, Department of Environmental Genomics, School of Public Health, Collaborative Innovation Center for Cancer Personalized Medicine, Nanjing Medical University, Nanjing, China; 2 The Key Laboratory of Modern Toxicology of Ministry of Education, Department of Genetic Toxicology, School of Public Health, Center for Global Health, Nanjing Medical University, Nanjing, China; 3 Cancer Epidemiology Division, Population Sciences in the Pacific Program, University of Hawaii Cancer Center, University of Hawaii at Manoa, Honolulu, HI USA; 4 Department of Bioinformatics, School of Biomedical Engineering and Informatics, Nanjing Medical University, Nanjing, China; 5 Department of Ophthalmology, Shanghai General Hospital, Shanghai Jiao Tong University School of Medicine, Shanghai, 200080 China; 6 Department of Biostatistics, School of Public Health, Center for Global Health, Nanjing Medical University, Nanjing, China; 7 Gusu School, The Affiliated Suzhou Hospital of Nanjing Medical University, Suzhou Municipal Hospital, Nanjing Medical University, Suzhou, China; 8 Department of Urology, The First Affiliated Hospital of Nanjing Medical University & Jiangsu Province People’s Hospital, 300 Guangzhou Road, Nanjing, 210029 China; 9 Department of Environmental Health, Harvard T.H. Chan School of Public Health, Harvard University, 655 Huntington Avenue, Boston, MA 02115 USA

**Keywords:** Age-specific genome-wide association studies, Early-onset prostate cancer, Phenome-wide association studies, Polygenic risk score, UK biobank

## Abstract

**Background:**

Early-onset prostate cancer (EOPC, ≤ 55 years) has a unique clinical entity harboring high genetic risk, but the majority of EOPC patients still substantial opportunity to be early-detected thus suffering an unfavorable prognosis. A refined understanding of age-based polygenic risk score (PRS) for prostate cancer (PCa) would be essential for personalized risk stratification.

**Methods:**

We included 167,517 male participants [4882 cases including 205 EOPC and 4677 late-onset PCa (LOPC)] from UK Biobank. A General-, an EOPC- and an LOPC-PRS were derived from age-specific genome-wide association studies. Weighted Cox proportional hazard models were applied to estimate the risk of PCa associated with PRSs. The discriminatory capability of PRSs were validated using time-dependent receiver operating characteristic (ROC) curves with additional 4238 males from PLCO and TCGA. Phenome-wide association studies underlying Mendelian Randomization were conducted to discover EOPC linking phenotypes.

**Results:**

The 269-PRS calculated via well-established risk variants was more strongly associated with risk of EOPC [hazard ratio (HR) = 2.35, 95% confidence interval (CI) 1.99–2.78] than LOPC (HR = 1.95, 95% CI 1.89–2.01; *I*^*2*^ = 79%). EOPC-PRS was dramatically related to EOPC risk (HR = 4.70, 95% CI 3.98–5.54) but not to LOPC (HR = 0.98, 95% CI 0.96–1.01), while LOPC-PRS had similar risk estimates for EOPC and LOPC (*I*^*2*^ = 0%). Particularly, EOPC-PRS performed optimal discriminatory capability for EOPC (area under the ROC = 0.613). Among the phenomic factors to PCa deposited in the platform of *ProAP* (***Pro****state cancer* ***A****ge-based* ***P****heWAS*; https://mulongdu.shinyapps.io/proap), EOPC was preferentially associated with PCa family history while LOPC was prone to environmental and lifestyles exposures.

**Conclusions:**

This study comprehensively profiled the distinct genetic and phenotypic architecture of EOPC. The EOPC-PRS may optimize risk estimate of PCa in young males, particularly those without family history, thus providing guidance for precision population stratification.

**Supplementary Information:**

The online version contains supplementary material available at 10.1186/s12967-024-05190-y.

## Introduction

Prostate cancer (PCa) is the most common malignancy in men in the Western world [1]. Established risk factors of PCa includes race, older age, and positive family history (FH) [2]. A positive FH of PCa is associated with a two- to threefold greater risk of PCa [3]. Notably, such effect is not uniform across ages; positive FH was shown to be associated with a higher risk increase among younger men compared with older men [4–6]. In the late 1990s and early 2000s, the incidence of PCa among young men experienced a disproportionate increase [7]. However, early-onset Prostate Cancer (EOPC) as a unique clinical entity remains insufficiently characterized [8].

Recently, Conti et al*.* performed a trans-ancestry (European, African, East, Asian and Hispanic) genome-wide association meta-analysis which led to the identification of 269 common risk variants for PCa [9]. In this work it was observed that the polygenic score (PRS) derived from these variants were more strongly associated with EOPC than with late-onset PCa (LOPC). In addition, early-onset patients tend to possess larger number of disease-related genetic variants [10]. Based on the enrichment of genetic risk factors in EOPC, we hypothesized that previous genome-wide association studies (GWAS) including subjects of all age groups underestimated the effect sizes of risk loci specific for EOPC. Study concentrating on younger population would profoundly reveal the genetic architecture of EOPC and refine the comprehension of age-based PRS for PCa.

In this study, we evaluated the genetic architecture of EOPC against LOPC and developed an EOPC-specific PRS based on a large-scale UK biobank cohort, accompanied by the validation from the Prostate, Lung, Colorectal and Ovarian (PLCO) cohort and The Cancer Genome Atlas (TCGA) program. We further performed a phenome-wide association study (PheWAS) to discover EOPC linking phenotypes via Mendelian Randomization (MR) analyses. This study discovered the distinct genetic and phenomic characteristics of EOPC, and proposed age-of-onset specific PRSs to optimize the risk estimate of prostate cancer.

## Materials and methods

### Study population and outcome ascertainment

The UK Biobank cohort recruited 502,528 participants aged 40–69 years between 2006 and 2010 [11]. The quality control of population was described in our previous study [12]. In brief, we developed a cancer follow-up cohort containing 355,543 participants met the following criteria: (1) without prevalent cancer at baseline (except non-melanoma skin cancer), (2) without sex discordance, (3) without outliers for genotype missingness or excess heterozygosity, (4) decided to participate in this program, (5) were self-reported as “white British” and genetically confirmed European ancestry, and (6) were unrelated individuals.

Incidence of PCa was defined based on the International Classification of Diseases, 10th revision (ICD-10) code C61. Participants were followed up from the enrolment until the time of PCa diagnosis or censoring (death, withdrawal or end of follow-up), at which the age named as exit-age. We developed a General-population covering all males (167,517 with 4882 incident PCa cases), an EO-population comprising individuals with exit-aged ≤ 55 years (28,725 with 205 cases), and an LO-population with exit-aged > 55 years (138,792 with 4677 cases). Participants in each population were separated into positive and negative first-degree FH group according to the self-reported prostate cancer of father or brothers.

The validation population was consisted of European ancestry participants from the PLCO cohort and TCGA program (permissions from dbGaP: Project #32547). The genotyping and imputation process have been described in our previous studies [13, 14]. Outlier individuals were removed using *smartpca* function from *EIGENSOFT* (version 5.0.2). Then the top ten principal components of retained individuals were calculated in company with the 1000 Genomes Project to determine the similarity of the genetic structure. Among the retained individuals, we randomly selected 1038 from 4974 LOPC cases to meet the ratio of EOPC against LOPC in UK biobank, and eventually constructed a population consisting of 87 EOPC, 1038 LOPC and 3113 controls.

### Risk variants identification and PRS calculation

We extracted 269 well-established risk variants for PCa from a trans-ancestry meta-analysis study (Additional file 2: Table S1) [9]. After excluding variants on the sex chromosome, SNPs and corresponding effect size in the European population were used to calculate a weighted PRS (named 269-PRS) as followed: for an individual *j*, ${PRS}_{j}=\sum_{i=1}^{N}{\beta }_{i}\times {SNP}_{ij}$, in which *i* represents each variant, ${\beta }_{i}$ equals to ${\text{ln}}(OR)$, $N$ is the total number of included variants, and ${SNP}_{ij}$ is the number of risk alleles (0, 1, 2) carried by variant *i* for individual *j*.

Next, we performed GWAS for General-, EO-, and LO-population, respectively. The quality control criteria were applied for each population: minor allele frequency ≥ 0.01, Hardy–Weinberg equilibrium *P*-value ≥ 1 × 10^–6^, and call rate > 95%. Genome-wide Cox regression analyses were conducted to identify risk variants with a suggestive threshold *P*-values < 10^–5^. Then, we used UK biobank (white British) data for pairwise linkage disequilibrium (LD) analysis to identify “independent variants” under *r*^*2*^ < 0.8.

As described previously, General-PRS, EOPC-PRS, and LOPC-PRS were developed using the independent variants and the corresponding *β*-value across each population with the formula: ${PRS}_{j}=\sum_{i=1}^{N}{\beta }_{i}\times {SNP}_{ij}$, where *i* represents variant, $N$ equals to the total number of included variants, and ${Dos}_{ij}$ is the genotype dosage for individual *j* for variant *i*. All PRSs were divided into quintiles based on the distribution among the general population for further analyses.

### Weighted Cox proportional hazard (WCoxPH) model

We used age-specific PCa incidence rates (termed as *inc*_*t*_) to create weights for Cox proportional hazards models. The cancer rates were reported for 5-year intervals from the CDC US Cancer Statistics (https://www.cdc.gov/cancer/uscs/) for multi-ancestries from 1999 to 2019. We used the incidence rate of White men to represent the rate of European ancestry men in this study. Linear interpolation was used to determine the rate for each year of age from 32 to 87. WCoxPH models was applied to estimate the risk of PCa associated with PRSs through assigning weights of 1 to cases and weights of 1/*inc*_*t*_ to non-cases using R package *survey* [15].

### Sensitivity analyses

Firstly, we re-analyzed the association of PRSs with incident and prevalent PCa using logistic regression models in a case–control population comprising 378,487 individuals, from which a General-, an EO- and an LO-group were developed according the reference-age (age at diagnosis of PCa or censoring) [16]. The case–control population was developed under the same quality control criteria as the aforementioned for follow-up cohort except for that (1) without prevalent cancer at baseline (except non-melanoma skin cancer). Secondly, aiming at increasing the power of PRSs, we merged well-established variants with GWAS independent ones across General-, EO- and LO-population then developed a merged-General-, a merged-EOPC-, and a merged-LOPC-PRS, respectively. GWAS independent variants were replaced by reported ones with *r*^*2*^ ≥ 0.8. The *β*-values were represented by ${\text{ln}}(\text{reported}\; \text{OR})$ for reported variants and the identified *β*-value for independent ones. The risk of PCa were re-estimated with the merged-PRSs.

### Validation of discriminatory capabilities for prediction

We introduced the receiver operating characteristic (ROC) curve (AUC) to evaluate the discriminatory accuracy for prediction of the aforementioned PRSs the validation population. Time-dependent ROC curves were plotted with *survivalROC* package from censored diagnosis data at 65-year, 70-year (cases diagnosed at 55 or younger removed), and 85-year (cases diagnosed at 70 or younger removed), regarding the date at birth as the beginning of follow-up, with Kaplan–Meier (KM) as the method for fitting joint distribution of risk-time [17].

### PheWAS for PCa

We performed pheWAS underlying two-sample MR analysis using R package *TwoSampleMR* to link causal traits to PCa, EOPC and LOPC [18]. There were 42,333 traits accessible in the IEU GWAS database up to July 2022; after excluding traits only concerning females and not originated from European population, we included 37,838 traits for analysis. Clumping was applied to select instrumental variables (IV). Clumps were performed around the central “index variants”, which was chosen with *P*-value < 5 × 10^–8^ and starting with the lowest *P*-value. Secondary hits were identified if they (a) were within the clumping window (10 Mb) of an index variant, (b) reached GWAS significance (*P*-value < 5 × 10^–8^), and (c) had a low LD with the index variants (*r*^*2*^ < 0.001 based on the 1000 Genomes Project European reference). Two-sample MR analyses were performed via inverse-variance weighted (IVW) and weighted media [18]. We also performed heterogeneity analyses to test whether IVs are related to other confounding factors, and MR-egger regression to assess the horizontal pleiotropy. High-confidence estimates should meet the following criteria: (1) IVW and weighted median estimates were directionally concordant with *P*-values < 0.05, and (2) *P*-value of heterogeneity and MR-egger regression analyses ≥ 0.05. Two-sample MR analyses were carried out with R package *TwoSampleMR* (v.0.4.26). Deposition of all MR results were generated using R package *shiny*.

### Statistical analyses

Statistical analyses were conducted with PLINK (version 1.90) and R (version 4.0.5). Risk estimates for PCa associated with PRSs were carried out using WCoxPH models for follow-up cohort and logistic regression models for case–control population. WCoxPH models were adjusted for covariates including age at assessment, assessment center, and top 10 principal components. For logistic regression models, we adjusted for reference-age, assessment center and top 10 principal components.

## Results

### 269-PRS is more strongly associated with EOPC

The process of this study was illustrated in Additional file 1: Fig. S1. A total of 167,517 individuals (4882 incident PCa cases) were retained to develop the General-population, which was separated to the EO-population with 28,725 participants (205 incident PCa cases) and the LO-population including 138,792 (4677 incident PCa cases; Additional file 1: Fig. S2A). The baseline characteristics for three populations are shown in Table 1. The age-specific PCa incidence rates were visualized in Additional file 1: Fig. S3, which were used for the sampling weighting in the Weighted Cox proportional hazard (WCoxPH) models.

**Table 1:** Baseline characteristics for males from UK biobank cancer follow-up cohort

Follow-up cohort	General-population		EO-population		LO-population	
	(N = 167,517)		(N = 28,725)		(N = 138,792)	
	Non incident	Incident PCa	Non incident	Incident PCa	Non incident	Incident PCa
N (%)	162,635 (97.09)	4882 (2.91)	28,520 (99.28)	205 (0.72)	134,115 (96.63)	4677 (3.37)
Age, Mean (SD)	56.83 (8.06)	62.37 (5.16)	44.07 (2.38)	49.05 (3.31)	59.54 (5.97)	62.95 (4.38)
Exit-age, Mean (SD)^a^	64.55 (8.03)	66.23 (5.32)	51.77 (2.30)	52.37 (2.64)	67.26 (5.91)	66.83 (4.53)
BMI, Mean (SD)	27.86 (4.25)	27.55 (3.83)	27.56 (4.29)	27.18 (3.53)	27.92 (4.23)	27.56 (3.84)
Smoking status, N (%)						
Never	79,849 (49.10)	2305 (47.21)	16,886 (59.21)	139 (67.80)	62,963 (46.95)	2166 (46.31)
Ever	82,238 (50.56)	2557 (52.38)	11,587 (40.63)	66 (32.20)	70,651 (52.68)	2491 (53.26)
Missing	548 (0.34)	20 (0.1)	47 (0.16)	0 (0.00)	501 (0.37)	20 (0.43)
Drinking status, N (%)						
Never	2698 (1.66)	78 (1.60)	410 (1.44)	4 (1.95)	2288 (1.71)	74 (1.58)
Ever	159,791 (98.25)	4804 (98.40)	28,080 (98.46)	201 (98.05)	131,711 (98.21)	4603 (98.42)
Missing	146 (0.09)	0 (0.00)	30 (0.11)	0 (0.00)	116 (0.09)	0 (0.00)
Family history of PCa, N (%)						
No	143,742 (88.38)	4034 (82.63)	25,548 (89.58)	153 (74.63)	118,194 (88.13)	3881 (82.98)
Yes	12,095 (7.44)	628 (12.86)	1858 (6.51)	44 (21.46)	10,237 (7.63)	584 (12.49)
Missing	6798 (4.18)	220 (4.51)	1114 (3.91)	8 (3.90)	5684 (4.24)	212 (4.53)

General-population covered all participants; EO-cohort was consisted of individuals with exit-age ≤ 55 years old; LO-cohort comprised individuals with exit-age > 55 years old

*PCa* prostate cancer, *PRS* polygenic risk score, *SD* standard deviation, *IQR* interquartile range

^a^Age for diagnosis of PCa or censoring otherwise

**Fig. 1:**
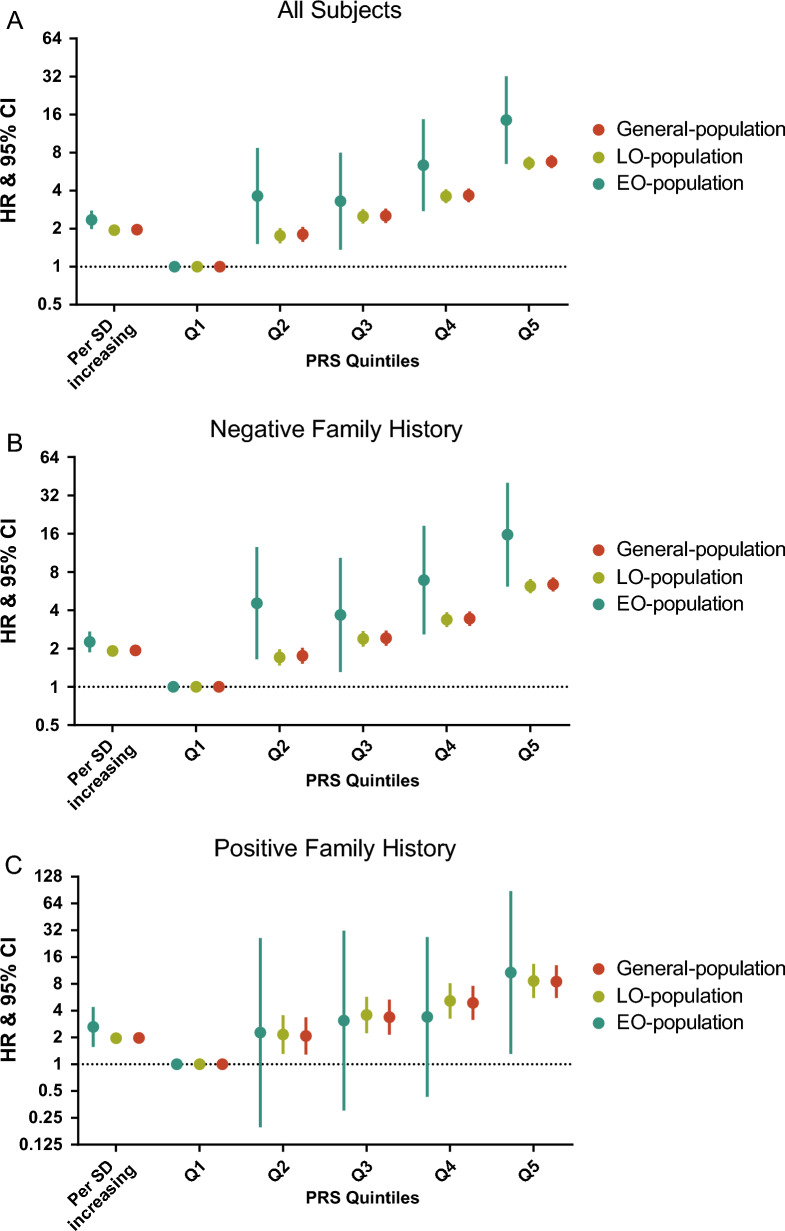
Risk estimates for PCa associated with a 269-PRS in the General-, EO- and LO-population. **A** Weighted Cox proportional hazard models include all subjects regardless of the family history of PCa. **B** Weighted Cox proportional hazard models include participants without a family history of PCa. **C** Weighted Cox proportional hazard models include participants with a family history of PCa. Models were adjusted for age at assessment, BMI, smoking status, drinking status, assessment center and top 10 principal components. General-population covered all participants; EO-population was consisted of individuals with exit-age ≤ 55 years old; LO-population comprised individuals with exit-age > 55 years old. *PCa* prostate cancer, *PRS* polygenic risk score, *HR* hazard ratio, *EO* early-onset, *LO* late-onset, *SD* standard deviation

We firstly assessed the risk of PCa associated with a 269-PRS derived from well-established risk variants (Additional file 2: Table S1) using a piece-wise WCoxPH model. There was a decreasing trend of the hazard ratio (HR) with age, ranking from 2.35 (1.99–2.78) in 40–55 to 1.84 (1.72–1.96) in 70–82 years old group [*I*^*2*^ = 63%, *P* for heterogeneity (*P*_*het*_) = 0.027; Additional file 1: Fig. S4]. In the subsequent division by age, we found that association with PCa risk per standard deviation of PRS in EOPC-population [HR = 2.35, 95% confidence interval (CI) 1.99–2.78] was stronger than that in LOPC-cohort (HR = 1.95, 95% CI 1.89–2.01; Fig. 1A and Additional file 2: Table S2), along with the significant heterogeneity (*I*^*2*^ = 79%, *P*_*het*_ = 0.031; Additional file 1: Fig. S5). We then performed stratified analysis by family history and found that PRS-associated risk for EOPC was stronger than that for LOPC mainly in participants with negative family history (EOPC: HR = 2.26, 95% CI 1.87–2.72; LOPC: HR = 1.92, 95% CI 1.85–1.98; *I*^*2*^ = 65%, *P*_*het*_ = 0.093; Fig. 1B, Additional file 1: Fig. S5 and Additional file 2: Table S2) but slightly in those with positive family history (EOPC: HR = 2.63, 95% CI 1.57–4.41; LOPC: HR = 1.97, 95% CI 1.81–2.14; *I*^*2*^ = 15%, *P*_*het*_ = 0.278; Fig. 1C, Additional file 1: Fig. S5 and Additional file 2: Table S2).

We then re-analyzed the association in the case–control population (Additional file 1: Fig. S2B and Additional file 2: Table S3). Consistently, the associations of 269-PRS with EOPC was stronger than that with LOPC [EOPC: odds ratio (OR) = 2.35, 95% CI 2.16–2.54; LOPC: OR = 1.96, 95% CI 1.91–2.01; *I*^*2*^ = 94%, *P*_*het*_ < 0.001], both in FH stratifications (Additional file 1: Figs. S6, S7 and Additional file 2: Table S4).

### EOPC harbors a specific genetic architecture

In the age-specific GWAS across General-, EO-, and LO-population, a total of 8,647,579, 8,676,601 and 8,652,442 available variants were included for Cox regression analyses (Additional file 1: Fig. S8). Totally, we identified 319, 45 and 296 independent loci for PCa, EOPC and LOPC after clumping, respectively (Additional file 2: Tables S5–S10), which were subsequently used for PRSs calculation. PCa and LOPC shared numerous common loci for PCa risk including 3q21.3, 8q24.21, 11q13.3, 17q24.3 and 19q13. We identified two clumps located on 6p22 specific for LOPC, represented by rs9404937 on 6p22.1 and rs9263530 on 6p22.33. As for EOPC, several notably distinct loci were observed, including 3q25.31, 8q13.1, 17q21.33 and 21q21.1.

**Fig. 2:**
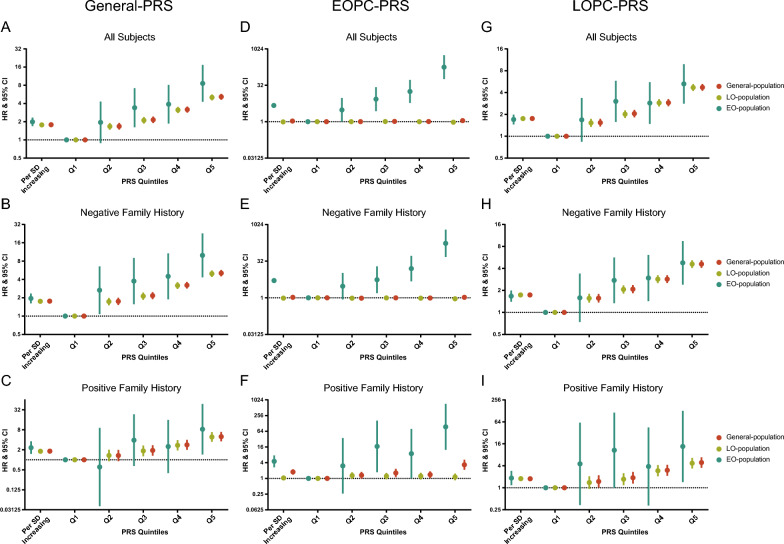
Risk estimates for PCa associated with a General-PRS (**A**–**C**), an EOPC-PRS (**D**–**F**) and an LOPC-PRS (**G**–**I**) in the General-, EO- and LO-population stratified by family history. Weighted Cox proportional hazard models were adjusted for age at assessment, BMI, smoking status, drinking status, assessment center and top 10 principal components. General-population covered all participants; EO-population was consisted of individuals with exit-age ≤ 55 years old; LO-population comprised individuals with exit-age > 55 years old. *PCa* prostate cancer, *PRS* polygenic risk score, *HR* hazard ratio, *EOPC* early-onset prostate cancer, *LOPC* late-onset prostate cancer, *SD* standard deviation

The associations of General-PRS with EOPC, similar to 269-PRS, was stronger than that with LOPC (EOPC: HR = 1.99, 95% CI 1.69–2.34; LOPC: HR = 1.77, 95% CI 1.72–1.83; *I*^*2*^ = 47%, *P*_*het*_ = 0.170], though such difference was not that obvious in the FH stratifications (Fig. 2A–C, Additional file 1: Fig. S9A and Additional file 2: Table S11). The EOPC-PRS demonstrated significant but weak association with the risk of PCa in the General-population (HR = 1.06, 95% CI 1.03–1.10; Fig. 2D and Additional file 2: Table S11]. Noteworthily, on contrary to the strong association with EOPC risk (HR = 4.70, 95% CI 3.98–5.54), the EOPC-PRS was not in relation to the risk of LOPC (HR = 0.98, CI: 0.96–1.01; *I*^*2*^ = 100%, *P*_*het*_ < 0.001), both in the FH stratifications (Fig. 2D–F, Additional file 1: Fig. S9B and Additional file 2: Table S11), implying the distinct genetic architecture of EOPC compared with LOPC. Furthermore, EOPC-PRS was strongly associated with risk of EOPC both in males without (HR = 5.12, 95% CI 4.23–6.20, *P* < 0.001) and with (HR = 7.00, 95% CI 3.93–12.47, *P* < 0.001) family history (Fig. 2E, F and Additional file 2: Table S11). Meanwhile, there was no heterogeneity between the risk estimates of EOPC and LOPC associated with the LOPC-PRS (EOPC: HR = 1.70, 95% CI 1.46–1.99; LOPC: HR = 1.75, 95% CI 1.70–1.81; *I*^*2*^ = 0%, *P*_*het*_ = 0.709), consistent in FH subgroups (Fig. 2G–I, Additional file 1: Fig. S9C and Additional file 2: Table S11).

**Fig. 3:**
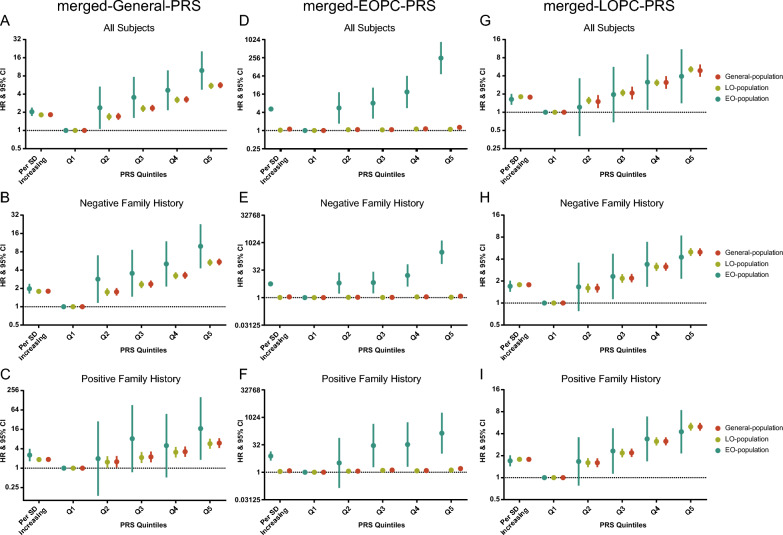
Risk estimates for PCa associated with a merged-General-PRS (**A**–**C**), a merged-EOPC-PRS (**D**–**F**) and a merged-LOPC-PRS (**G**–**I**) in the General-, EO- and LO-population stratified by family history. Weighted Cox proportional hazard models were adjusted for age at assessment, BMI, smoking status, drinking status, assessment center and top 10 principal components. General-population covered all participants; EO-population was consisted of individuals with exit-age ≤ 55 years old; LO-population comprised individuals with exit-age > 55 years old. *PCa* prostate cancer, *PRS* polygenic risk score, *HR* hazard ratio, *EOPC* early-onset prostate cancer, *LOPC* late-onset prostate cancer, *SD* standard deviation

The sensitivity analyses with case–control population yielded similar results (Additional file 1: Figs. S10, S11 and Additional file 2: Table S12). Next, we merged reported variants and GWAS independent ones for re-analyses (process illustrated in Additional file 1: Fig. S12); resultantly, 397, 62 and 375 variants were applied to build merged-General-, merged-EOPC- and merged-LOPC-PRS, respectively (Additional file 2: Tables S13–S15). Consistently, the association between merged-General-PRS and EOPC was stronger than that of LOPC (EOPC: HR = 2.04, 95% CI 1.74–2.39; LOPC: HR = 1.81, 95% CI 1.76–1.87; *I*^*2*^ = 49%, *P*_*het*_ = 0.162), which was more obviously in the negative FH (*I*^*2*^ = 48%, *P*_*het*_ = 0.167) than that in the positive family history group (*I*^*2*^ = 9%, *P*_*het*_ = 0.294; Fig. 3A–C, Additional file 1: Fig. S13A and Additional file 2: Table S16). The merged-EOPC-PRS was in a weak association with the risk of LOPC (HR 1.03, CI 1.00–1.06) but strong with EOPC (HR 5.16, CI 4.37–6.08; *I*^*2*^ = 100%, *P*_*het*_ < 0.001; Fig. 3D–F, Additional file 1: Fig. S13B and Additional file 2: Table S16). Similar to LOPC-PRS, the association of merged-LOPC-PRS with PCa risk was approximate between the EO- and LO-population (EOPC: HR 1.75, 95% CI 1.49–2.04; LOPC: HR 1.80, 95% CI 1.75–1.86; *I*^*2*^ = 0%, *P*_*het*_ = 0.679), both in FH stratifications (Fig. 3G–I, Additional file 1: Fig. S13C and Additional file 2: Table S16). Logistic regression analyses based on the case–control population manifested same results (Additional file 1: Figs. S14, S15 and Additional file 2: Table S17).

### EO-PRS exhibits high discriminatory capabilities for EOPC

**Fig. 4:**
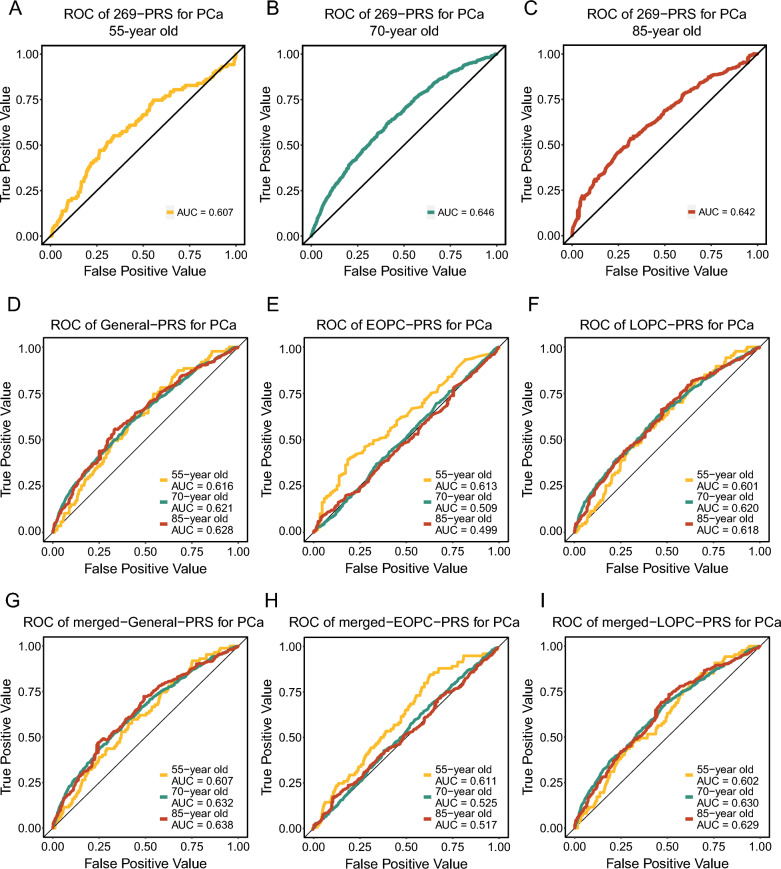
The area under the receiver operating characteristic (ROC) curve (AUC) evaluating the discriminatory accuracy for prediction of PRSs in a European ancestry population generated from the PLCO cohort and TCGA program. **A**–**C** Time-dependent ROC curves and AUCs from censored diagnosis data at 65-year (**A**), 70-year (cases diagnosed at 55 or younger removed) (**B**), and 85-year (cases diagnosed at 70 or younger removed) (**C**) of 269-PRS for prediction of PCa. **D**–**I** Time-dependent ROC curves and AUCs of General-PRS (**D**), EOPC- PRS (**E**), LOPC-PRS (**F**), merged-General-PRS (**G**), merged-EOPC-PRS (**H**) and merged-LOPC-PRS (**I**). *PLCO* Prostate, Lung, Colorectal and Ovarian; *TCGA* The Cancer Genome Atlas Program, *EOPC* early-onset prostate cancer, *LOPC* late-onset prostate cancer

We further test the prediction ability of EOPC-PRS in an additional European ancestry population (Additional file 1: Fig. S16). The discriminatory capabilities of 269-PRS and General-PRS for LOPC [70- and 85-year diagnostic AUC: 0.646 and 0.642 for 269-PRS; 0.621 and 0.628 for General-PRS] were better than that for EOPC (55-year AUC: 0.607 for 269-PRS; 0.616 for General-PRS, Fig. 4A–D). The EOPC-PRS harbored a distinctly high prediction capability for EOPC (55-year AUC = 0.613), compared with that for LOPC (70- and 85-year diagnostic AUC = 0.509 and 0.499); while LOPC-PRS had a higher discriminatory ability for LOPC (70- and 85-year diagnostic AUC = 0.620 and 0.618) but a lower one for EOPC (55-year AUC = 0.601, Fig. 4E, F). Consistent results were yielded for merged PRSs (Fig. 4G–I). We additionally collected two published PRSs associated with PCa risk that were derived from the general population and evaluated their predictive ability for EOPC [19, 20]. Comparison with these two PRSs, EOPC-PRS indeed demonstrates the optimal predictive capacity for EOPC (Additional file 1: Fig. S17). Moreover, the EOPC-PRS could refine the discriminatory capability of PSA in men before 60 years old (Additional file 1: Fig. S18) from PLCO cohort. We also found a significant correlation between EOPC-PRS and various clinical subgroups of prostate cancer that a stronger association was observed in early-stage EOPC (GS < 7, T stage ≤ T2 and N0; Additional file 1: Fig. S19). Therefore, these findings suggest that EOPC-PRS might be closely related with the risk of EOPC, especially those with early-stage tumors.

### EOPC is associated with unique phenomic features

**Fig. 5:**
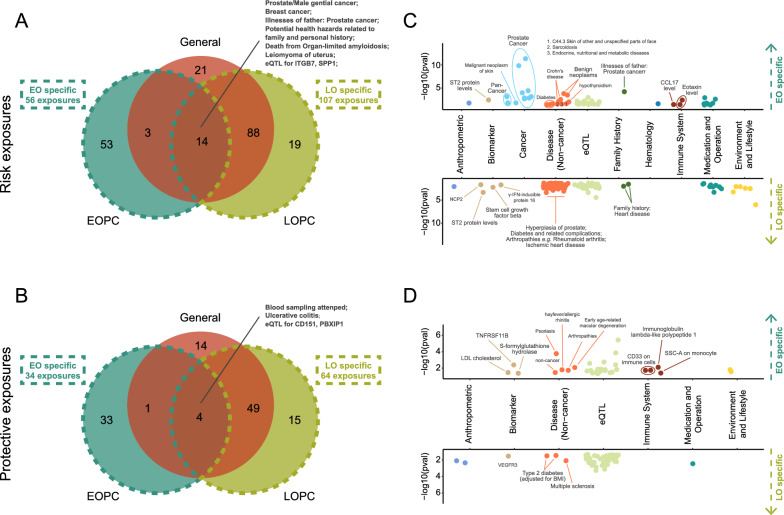
Phenome-wide association analysis of PCa, EOPC and LOPC through two-sample MR analyses based on UK Biobank GWAS summary data. **A** Venn diagram of high-confidence risk traits liked to PCa, EOPC and LOPC. **B** Scatter plots of EOPC specific (upper) and LOPC-specific (below) risk traits. **C** Venn diagram of high-confidence protective traits linked to PCa, EOPC and LOPC. **D** Scatter plots of EOPC specific (upper) and LOPC-specific (below) protective traits. *EOPC* early-onset prostate cancer, *LOPC* late-onset prostate cancer

To profile the traits potentially associated with EOPC and LOPC, we performed PheWAS via two-sample MR analysis (illustrated in Figure S20). We identified 194, 108 and 189 high-confidence traits for total PCa, EOPC and LOPC, respectively (Additional file 2: Tables S18–S20). Generally, 90 (56 risk and 34 protective) and 171 (107 risk and 64 protective) causal traits were specific to EOPC and LOPC, respectively, and 14 risk and 4 protective traits were shared among the three sets, that included PCa and PCa FH (Fig. 5A, B). Notably, eleven of 56 EOPC-specific risk traits were cancers, two were immune system, and one was another trait concerning PCa FH. On the contrary, among the 107 risk traits specific to LOPC, none of them were associated with cancer or immune system. LOPC was mainly linked to diseases in the aged including prostate hyperplasia, arthropathies, diabetes, etc. Noteworthily, multiple environmental/lifestyle traits (e.g., population density, alcohol intake) were dramatically associated with LOPC, but not EOPC (Fig. 5C). Besides, the protective traits specific to EOPC embracing LDL cholesterol and arthropathies, which was inverse to LOPC risk traits. (Fig. 5D). The above findings are deposited on a user-friendly online tool: ***Pro****state cancer* ***A****ge-based* ***P****heWAS* (*ProAP*, https://mulongdu.shinyapps.io/proap). The results can be focused on a certain outcome among “General-PCa”, “EOPC” and “LOPC” through a selection in the “Choose an outcome” box. Additionally, a trait of interest is easily accessible by using “Search” box. For instance, the user can choose “EOPC” and search “illness of father” to obtain the causal effects of family history of father on EOPC development (Additional file 1: Fig. S21).

## Discussion

In this study, we delineated a specific genetic architecture to EOPC and developed a 45-SNP-EOPC-PRS for PCa risk prediction with great discriminatory capabilities in younger men. Moreover, EOPC was discovered significantly linked to causal traits concerning genetic factors such as PCa family history while not to environmental or lifestyle traits.

PCa mainly occurred in the aged, accounting for only 8% under 55 years old [21]. Some studies reported that EOPC have a favorable prognosis, partly because younger patients have fewer comorbid conditions and better tolerance for more aggressive treatment [22, 23]. However, these clinical observations mainly focused on men who were eligible for therapies, and therefore probably ignored young men with high grade PCa [24]. However, population-based data suggested that the younger groups were at a higher risk of all-cause and cancer-specific death [24]. Also, according to the Surveillance, Epidemiology, and End Results Program database covering 48% of the U.S. population, the 5-year relative survival rate of PCa was 96.2% for patients diagnosed less than 50 years old, 98.2% for 50–64, and 99.2% for 65–74 [25]. In fact, the viewpoint that EOPC patients have poorer prognosis was widely spread among physicians in the pre-PSA era based on clinical observation [26, 27] and large-scale cohorts [28–30]. Additionally, patients with EOPC are less likely to die from other causes than those with LOPC [24]. So far, prostate specific antigen (PSA) testing is “informed select” only in elder men unless existing a FH [8]; however, only 21.5% of EOPC in our data had a FH. Therefore, the majority of EOPC still lacks substantial opportunity to be early-detected thus suffering an unfavorable prognosis. It is plausible that EOPC is a unique clinical entity which deserves more attention.

Compared with LOPC, EOPC participants harbored marked skewing towards a positive FH, and such fact limited the clinical usage of genetic models in diagnosis because of the readily accessibility of FH. Studies reported that the AUC only improved from 0.526 (FH alone) to 0.642 (genetic markers + FH) for the diagnosis of PCa [31]. Though, Mars et al*.* demonstrated that first-degree FH only explained averagely 3% of the effect of PRSs, and PRSs was useful in refining risk assessment of PCa even when FH is available [32]. Additionally, we noted that all developed PRSs still have considerable predictive discriminatory capabilities for FH-free EOPC. Taken together, applying genetic information to the prediction of EOPC, particularly in men without a FH may yield additional clinical benefits.

The cumulative burden of PCa risk variants was found more strongly associated with EOPC in this study, consistent with previous studies [9, 10, 15, 19, 20, 31]. However, because these PRS were not established based on the EO population, the EOPC-PRS exhibits superior predictive capability for EOPC compared to them. In addition to previous studies, we carried out age-specific GWAS for EOPC and LOPC. Different from the common loci (e.g., 3q21.3, 8q24.21, 11q13.3 [33–35]) associated with risk of total PCa and LOPC, EOPC was related to specific loci including 3q25.31, 8q13.1, 17q21.33 and 21q21.1. Besides, the EOPC-PRS having a strong association with EOPC risk but not with LOPC. Such distinct genetic pattern of EOPC was also validated in the sensitive analyses. In a study concerning early-onset colorectal cancer (EOCRC), it was suggested that there is still space for improving the discriminatory accuracy of PRS because the included SNPs were not specific to early-onset disease [16]. We believed that variants and EOPC-PRS produced from EOPC-specific GWAS were able to promote the predictive capability of EOPC. Further, in the association analysis stratified by clinical characteristics, we observed that EOPC-PRS effectively differentiated individuals with high risk of EOPC, especially early-stage tumors. Though may not in high risk to develop aggressive PCa, this population deserve early clinical management such as active surveillance. A previous study conducted a GWAS specific to EOPC, identifying two significant loci at 8q24 and 11p15 [36]. In comparison to this study, our study further placed a greater emphasis on constructing PRSs stratified by age, based on which we systematically illustrated the differences in the genetic architectures between EOPC and LOPC and highlighted the risk prediction value of these PRSs.

Studies have reported risk factors for PCa environmentally, habitually and biologically [37–39]*.* Environmental/lifestyle exposures influence the cancer risk in a cumulative pattern; the short period available for younger men to accrue these exposures is consistence with the higher genetic association in early-onset patients. In line with expectations, we found environmental/lifestyle traits had causal effects on LOPC, but not on EOPC. Additionally, LOPC was linked to traits encompassing ischemic heart diseases, diabetes, etc., which were related to comorbidities more common in elders. All results highlighted the uniqueness of phenomic characteristics of EOPC; identification of risk factors genetically and biologically could help in the risk stratification of EOPC in union with EOPC-PRS.

In this study, we applied a weighted Cox proportional hazards model to estimate the risk of PCa associated with PRSs, which was adopted by Schaid et al. in their study [15]. Studies have shown that applying Cox regression models with the usage of age information can lead to statistical power compared with logistic model [40]. Therefore, we estimated relative risks through a Cox proportional hazard model with sampling weights based on incidence rates to account for how cases and controls were sampled. To minimize the bias caused by overfitting, we developed PRSs from three sub-populations and carried out cross-validation, and then conducted two-stage sensitivity analyses, to enhance the convincingness of results. This study did have several limitations. Firstly, the size of EOPC population was relatively small, leading to an inadequate power for GWAS. Thus, we applied a suggestive GWAS threshold of 10^–5^ and a loose clumping criterion of 0.8 in order to bring in as many as loci to describe the genetic architecture of EOPC and improve the power of PRSs. Moreover, in the PheWAS section, we aimed at presenting a sketchy phenomic landscape; therefore, some exposures originated from UK biobank were uniformly analyzed via two-sample MR (one-sample MR used commonly), so as to adequately characterize the uniqueness of EOPC compared with LOPC. We did not calculate *F*-statistics for the identification of weak instruments because effect allele frequencies are not uniformly provided by the summary data of every exposure. According to our experience, a criterion with *P*-value < 5e−8 and clumping *r*^2^ < 0.001 rarely leads to weak instruments, because the *F*-statistics usually exceed 30. So, any bias introduced by weak instruments, if present at all, was exceedingly minimal. Lastly, because our study was based on the ‘white British’ population from the UK Biobank, the applicability of the PRSs identified in our study might be constricted to males of European ancestry. Further validations in other populations are needed to enhance their generalizability.

## Conclusions

This study comprehensively delineates the unique genetic architecture and risk factors of EOPC that were differed from LOPC. A newly developed EOPC-PRS with strongest and specific association with EOPC may optimize risk stratification of PCa in young males (particularly those without FH), and subsequently provide guidance for personalized interventions.

### Supplementary Information

**Additional file 1: Fig. S1.** Flowchart of this study. Exit-age: age at diagnosis or censoring. **Fig. S2.** Population ascertainment of the UK Biobank cohort. **Fig. S3.** Age-specific incidence of prostate cancer per 100,000 for White ancestry population from the CDC US Cancer Statistics. **Fig. S4.** Estimate risk for PCa associated a 269-PRS for age groups with weighted Cox proportional hazard models. **Fig. S5.** Forest plot of the heterogeneity analyses between the EOPC and LOPC risk measured by weighted Cox proportional hazard models. **Fig. S6.** Risk estimates for PCa associated with PRSs from the case–control population. **Fig. S7.** Forest plot of the heterogeneity analyses between the EOPC and LOPC risk. **Fig. S8.** Genome-wide association studies (GWAS) for PCa risk in General-population, EO-population and LO-population using Cox proportional hazard models. **Fig. S9.** Forest plot of the heterogeneity analyses between the EOPC and LOPC risk. **Fig. S10.** Risk estimates for PCa associated with PRSs from the case–control population. **Fig. S11.** Forest plot of the heterogeneity analyses between the EOPC and LOPC risk measured by logistic regression models. **Fig. S12.** Flowchart of merging reported variants and GWAS top variants. **Fig. S13.** Forest plot of the heterogeneity analyses between the EOPC and LOPC risk measured by weighted Cox proportional hazard models. **Fig. S14.** Risk estimates for PCa associated with PRSs from the case–control population. **Fig. S15.** Forest plot of the heterogeneity analyses between the EOPC and LOPC risk. **Fig. S16.** Population structure demonstrated by principal component analysis based on all high-quality SNPs. **Fig. S17.** The area under the receiver operating characteristic (ROC) curve (AUC) evaluating the predictive accuracy of EOPC-PRS (**A**), 54-PRS (**B**) and 110-PRS (**C**) for EOPC in a European ancestry population generated from the PLCO cohort and TCGA program. **Fig. S18.** Time-dependent receiver operating characteristic (ROC) curves and area under the curves (AUC) from censored diagnosis data at 60-year of PSA and PSA + EOPC-PRS for prediction of PCa. **Fig. S19.** Risk estimates for early-onset prostate cancer (EOPC) associated with EOPC-PRS stratified by clinical variables (Gleason score, T stage and M stage). **Fig. S20.** Flowchart of two-sample Mendelian randomization analyses. **Fig. S21.** An example for the use of ProAP (Prostate cancer Age-based PheWAS).

**Additional file 2: Table S1.** Prostate cancer association results for 269 established PCa risk variants from Conti et al. **Table S2.** Hazard ratio for PCa associated with a 269-SNP PRS. **Table S3.** Baseline characteristics for General-, EOPC- and LOPC-group from UK Biobank case–control population. **Table S4.** Odds ratio for PCa associated with a 269-SNP PRS. **Table S5.** 2,555 Genome-wide significant variants associated with the risk of PCa in the Cox proportional hazard model in UK Biobank. **Table S6.** 91 Genome-wide significant variants associated with the risk of EOPC in the Cox proportional hazard model in UK Biobank. **Table S7.** 2500 Genome-wide significant variants associated with the risk of LOPC in the Cox proportional hazard model in UK Biobank. **Table S8.** 319 independent variants among 1555 Genome-wide significant variants associated with the risk of PCa based on clumping in 1000 Genomes Project. **Table S9.** 45 independent variants among 223 Genome-wide significant variants associated with the risk of EOPC based on clumping in 1000 Genomes Project. **Table S10.** 296 independent variants among 1583 Genome-wide significant variants associated with the risk of LOPC based on clumping in 1000 Genomes Project. **Table S11.** Hazard ratio for PCa associated with the General-, EOPC- and LOPC-PRS based on UK BioBank follow-up cohort. **Table S12.** Odds ratio for PCa associated with the General-, EOPC- and LOPC-PRS based on UK BioBank case–control population. **Table S13.** 397 merged SNPs from the 269-SNP PRS and General-PRS. **Table S14.** 62 merged SNPs from the 269-SNP PRS and EOPC-PRS. **Table S15.** 375 merged SNPs from the 269-SNP PRS and LOPC-PRS. **Table S16.** Hazard ratio for PCa associated with the merged-General-, merged-EOPC- and merged-LOPC-PRS based on UK BioBank follow-up cohort. **Table S17.** Odds ratio for PCa associated with the merged-General-, merged-EOPC- and merged-LOPC-PRS based on UK BioBank case-comtrol population. **Table S18.** High-confidence traits linked to PCa through two-sample MR analysis. **Table S19.** High-confidence traits linked to EOPC through two-sample MR analysis. **Table S20.** High-confidence traits linked to LOPC through two-sample MR analysis.

## Data Availability

(1) The raw genotype and clinical data are accessible from UK Biobank (https://www.ukbiobank.ac.uk/) with approval (Application #45611). (2) The raw data of PLCO cohort are available from Genotypes and Phenotypes (dbGaP) accession phs000207.v1.p1 and phs000882.v1.p1 under permission (Project #32547). (3) The raw genotype and clinical data of TCGA are downloaded from dbGaP accession phs000178.v11.p8 under permission (Project #32547). (4) The summary statistics of Genome-wide Cox regression analyses and the key scripts of this study were deposited at https://github.com/ChengBioinfo/EOPC-PRS/tree/master.

## Abbreviations

EOPC: Early-onset prostate cancer
PRS: Polygenic risk score
PCa: Prostate cancer
LOPC: Late-onset prostate cancer
HR: Hazard ratio
CI: Confidence interval
PheWAS: Phenome-wide association study
ProAP: Prostate cancer age-based PheWAS
FH: Family history
GWAS: Genome-wide association studies
PLCO: Prostate, Lung, Colorectal and Ovarian
TCGA: The Cancer Genome Atlas
WCoxPH: Weighted Cox proportional hazard
ROC: Receiver operating characteristic
AUC: Area under the curve
IVW: Inverse-variance weighted
MR: Mendelian Randomization
OR: Odds ratio
SNP: Single nucleotide polymorphism
