# Supplementary material for: An early-onset specific polygenic risk score optimizes age-based risk estimate and stratification of prostate cancer: population-based cohort study
Source: J Transl Med. 2024 Apr 17;22:366. doi: 10.1186/s12967-024-05190-y (PMC11025178; doi:10.1186/s12967-024-05190-y)
Supplement: Supplementary file 1 — Additional file 1: Fig. S1. Flowchart of this study. Exit-age: age at diagnosis or censoring. Fig. S2. Population ascertainment of the UK Biobank cohort. Fig. S3. Age-specific incidence of prostate cancer per 100,000 for White ancestry population from the CDC US Cancer Statistics. Fig. S4. Estimate risk for PCa associated a 269-PRS for age groups with weighted Cox proportional hazard models. Fig. S5. Forest plot of the heterogeneity analyses between the EOPC and LOPC risk measured by weighted Cox proportional hazard models. Fig. S6. Risk estimates for PCa associated with PRSs from the case–control population. Fig. S7. Forest plot of the heterogeneity analyses between the EOPC and LOPC risk. Fig. S8. Genome-wide association studies (GWAS) for PCa risk in General-population, EO-population and LO-population using Cox proportional hazard models. Fig. S9. Forest plot of the heterogeneity analyses between the EOPC and LOPC risk. Fig. S10. Risk estimates for PCa associated with PRSs from the case–control population. Fig. S11. Forest plot of the heterogeneity analyses between the EOPC and LOPC risk measured by logistic regression models. Fig. S12. Flowchart of merging reported variants and GWAS top variants. Fig. S13. Forest plot of the heterogeneity analyses between the EOPC and LOPC risk measured by weighted Cox proportional hazard models. Fig. S14. Risk estimates for PCa associated with PRSs from the case–control population. Fig. S15. Forest plot of the heterogeneity analyses between the EOPC and LOPC risk. Fig. S16. Population structure demonstrated by principal component analysis based on all high-quality SNPs. Fig. S17. The area under the receiver operating characteristic (ROC) curve (AUC) evaluating the predictive accuracy of EOPC-PRS (A), 54-PRS (B) and 110-PRS (C) for EOPC in a European ancestry population generated from the PLCO cohort and TCGA program. Fig. S18. Time-dependent receiver operating characteristic (ROC) curves and area under the curves (AUC) from cen [file 12967_2024_5190_MOESM1_ESM.docx]

**Additional Figures:**

**Figure S1.** Flowchart of this study. Exit-age: age at diagnosis or censoring. ^a^ Detailed process of merging variants was shown in **Figure S2**. ^b^ Procedure of two-sample Mendelian randomization analysis was shown in **Figure S3**. PRS, polygenic risk score; MR, Mendelian randomization.


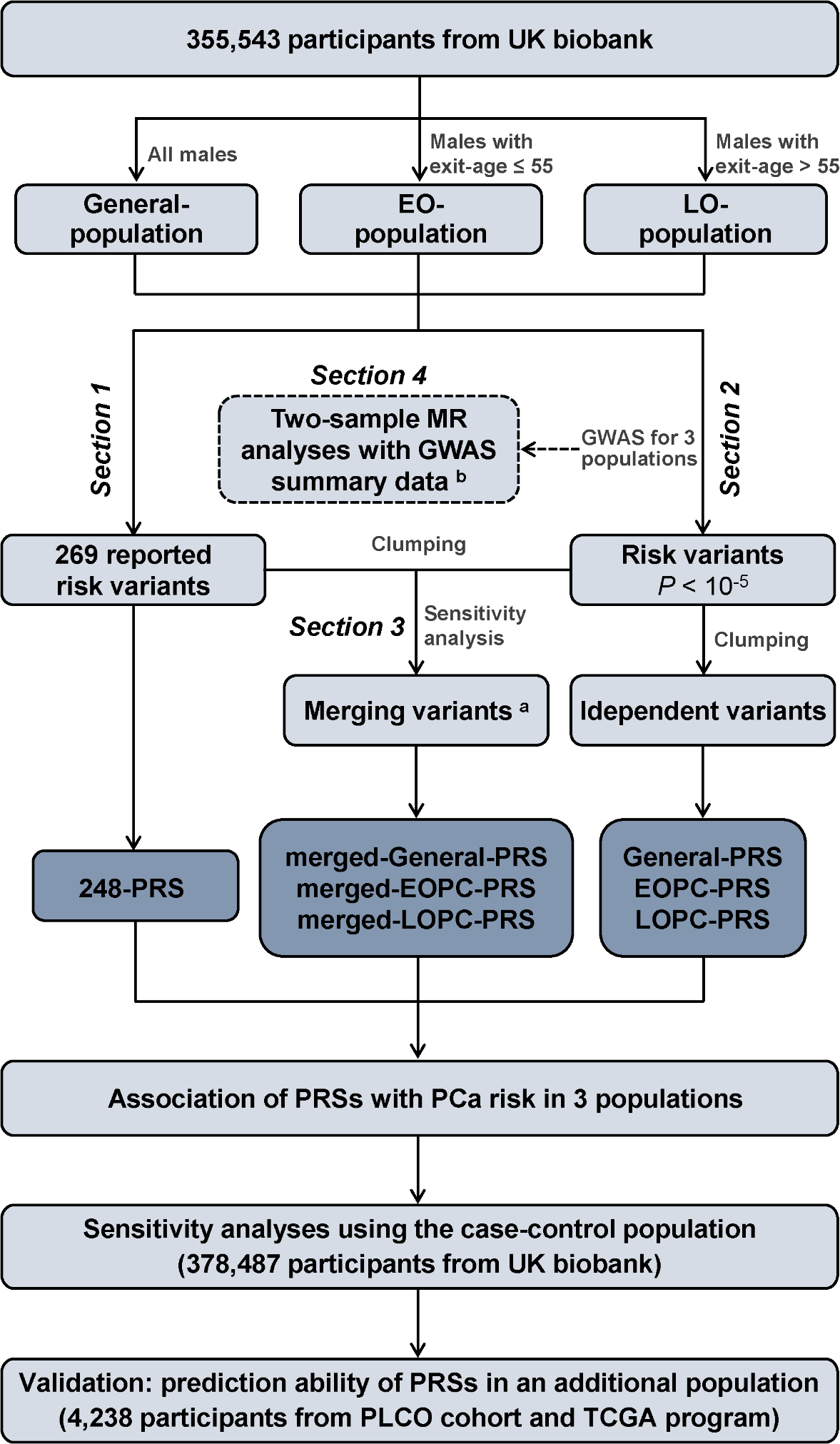


**Figure S2.** Population ascertainment of the UK Biobank cohort. (Upper) The cancer follow-up cohort comprising 167,517 individuals and was separated into an early-onset (EO)-population and a late-onset (LO)-population according to the exit-age. (Below) The case-control population comprising 175,349 males and was separated into an EO-group and an LO-group according to the reference-age.

**
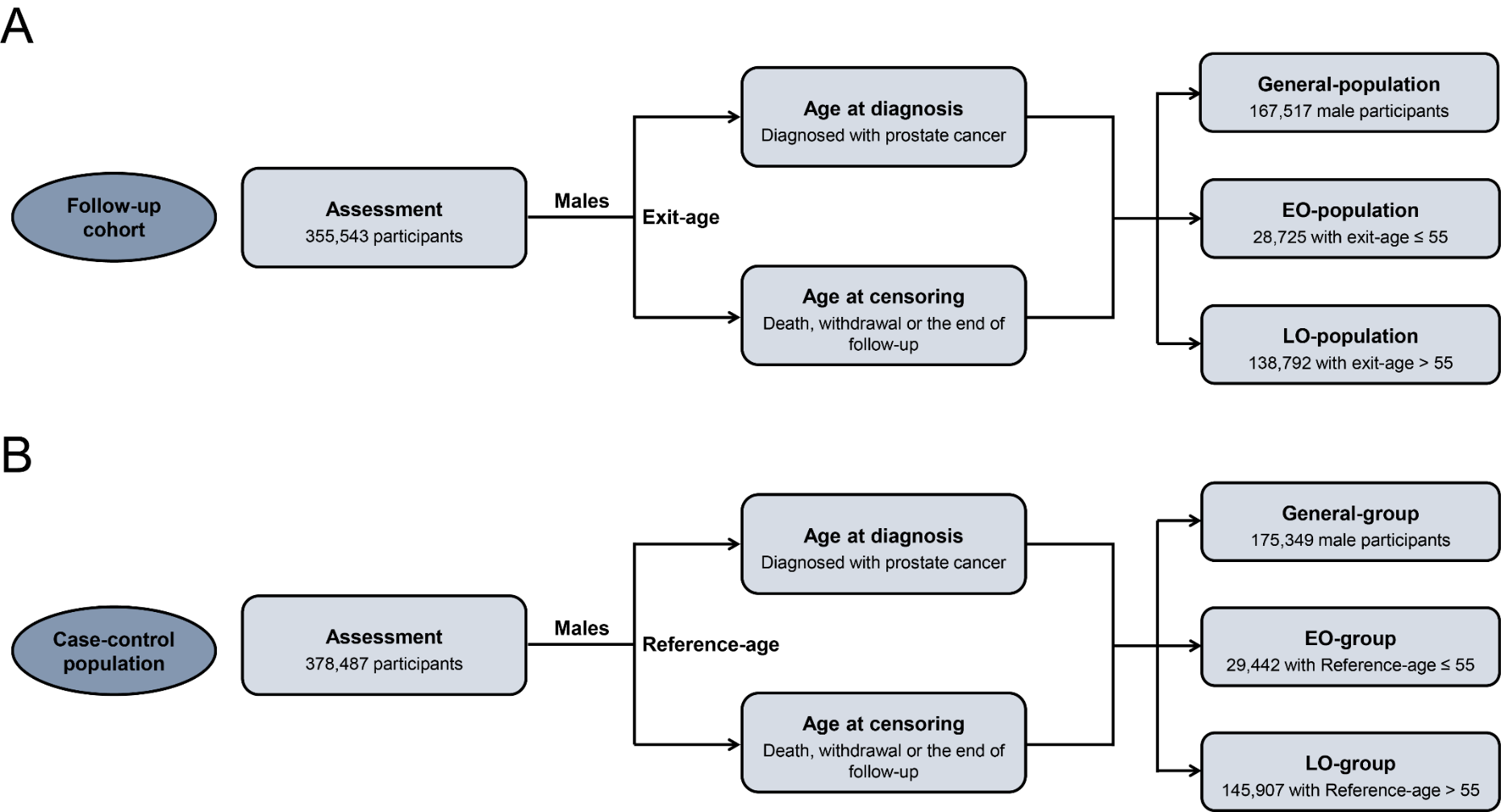
**

**Figure S3.** Age-specific incidence of prostate cancer per 100,000 for White ancestry population from the CDC US Cancer Statistics.

**
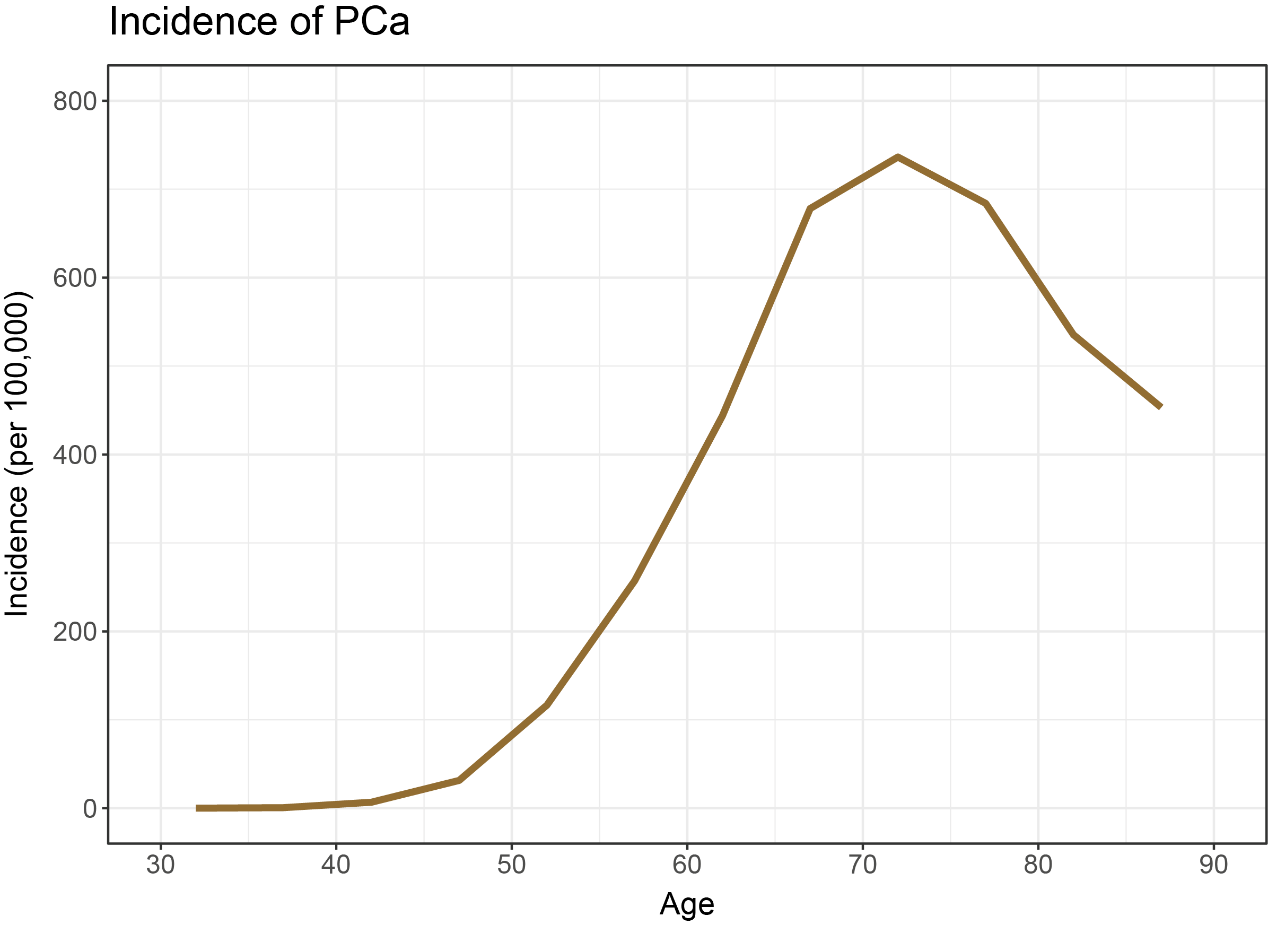
**

**Figure S4.** Estimate risk for PCa associated a 269-PRS for age groups with weighted Cox proportional hazard models. Models were adjusted for age at assessment, BMI, smoking status, drinking status, assessment center and top 10 principal components. PRS, polygenic risk score.


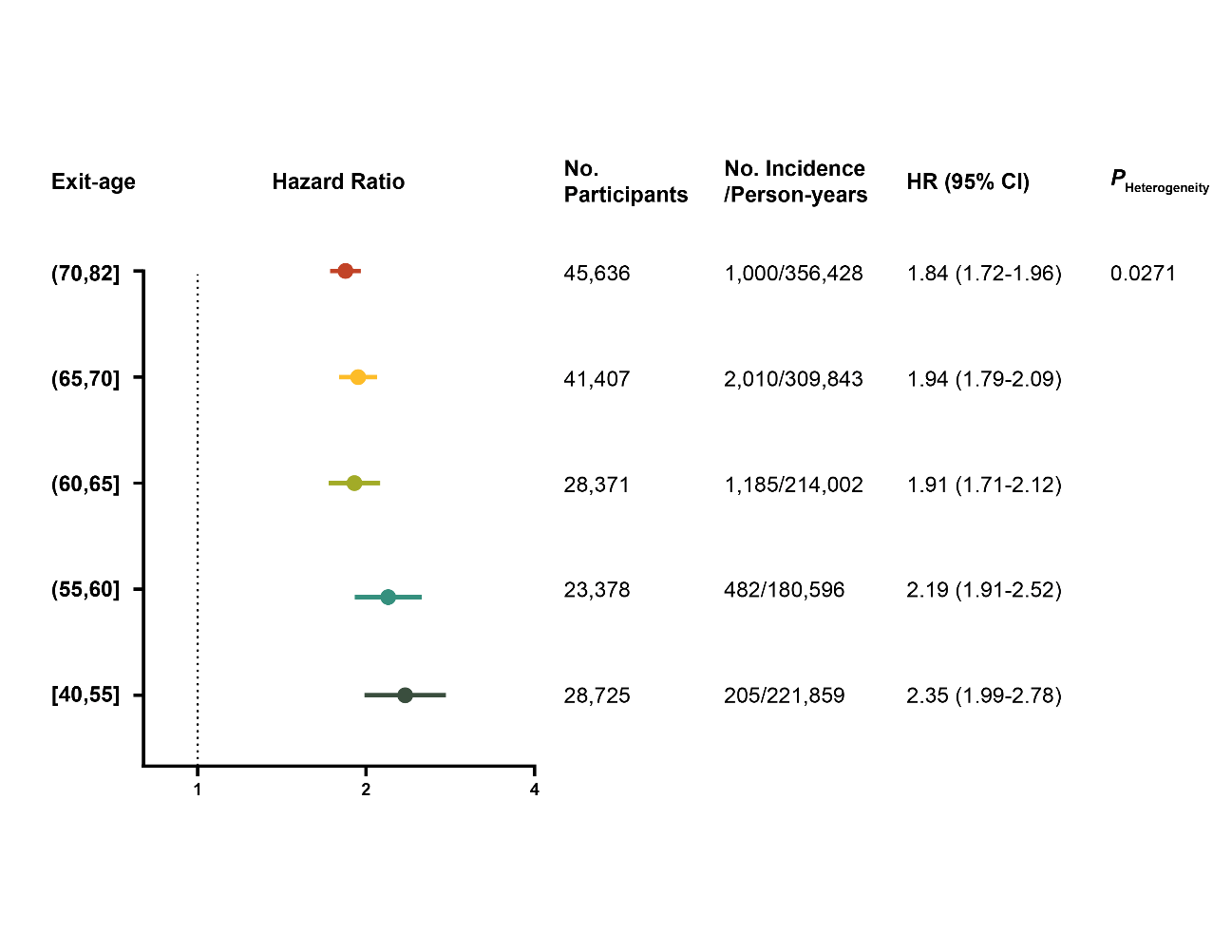


**Figure S5.** Forest plot of the heterogeneity analyses between the EOPC and LOPC risk measured by weighted Cox proportional hazard models associated with a 269-PRS stratified by family history. PRS, polygenic risk score; EOPC, early-onset prostate cancer; LOPC, late-onset prostate cancer; HR, hazard ratio; CI, confidence interval.


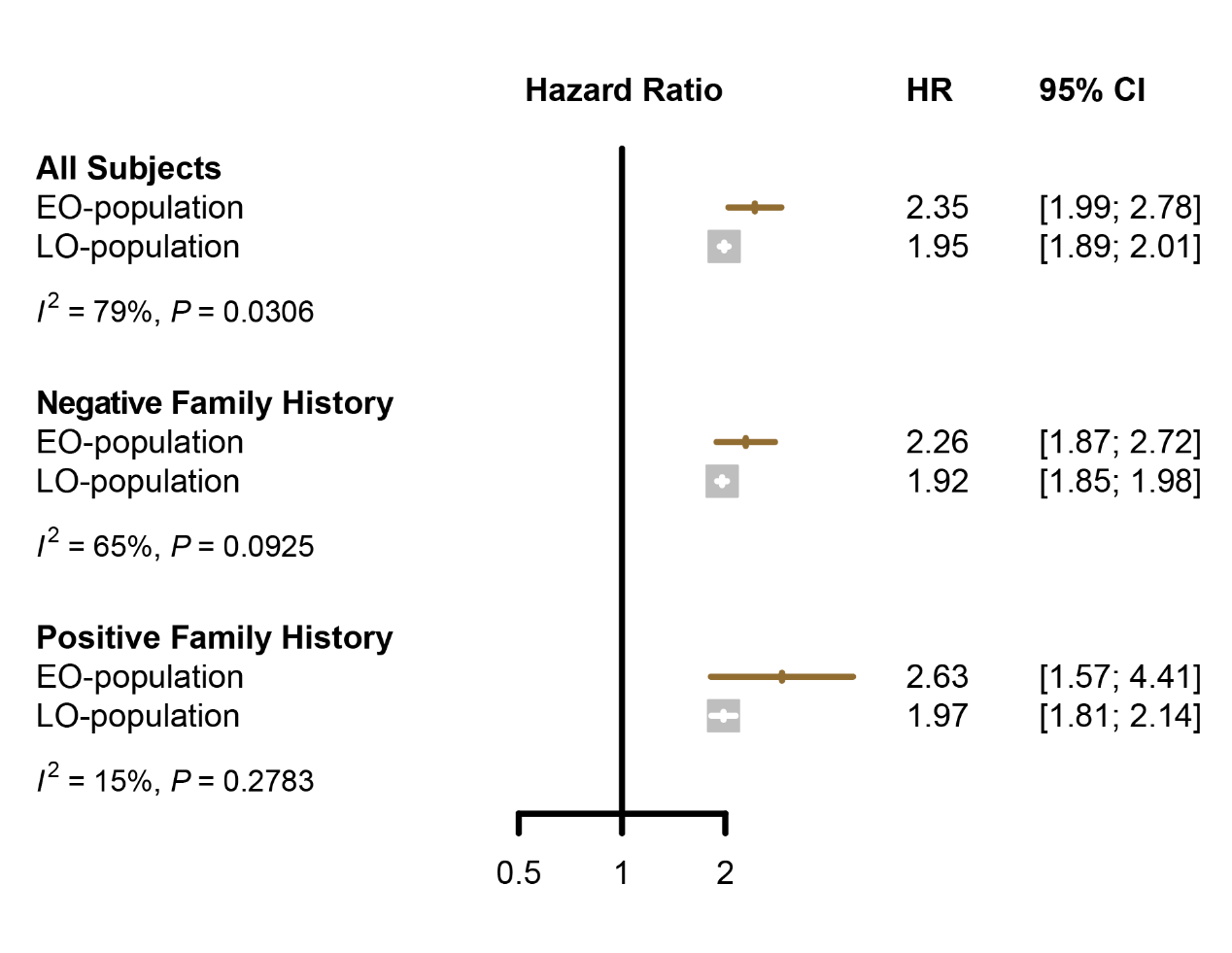


**Figure S6.** Risk estimates for PCa associated with a 269-PRS in the General-, EO- and LO-group from the case-control population. (A) Logistic regression models include all subjects regardless of the family history of PCa. (B) Logistic regression models include participants without a family history of PCa. (C) Logistic regression models include participants with a family history of PCa. Models were adjusted for reference-age, BMI, smoking status, drinking status, assessment center and top 10 principal components. General-group covered all participants; EO-group was consisted of individuals with reference-age ≤ 55 years old; LO-group comprised individuals with reference -age > 55 years old. PRS, polygenic risk score; EO, early-onset; LO, late-onset; OR, odds ratio; CI, confidence interval.


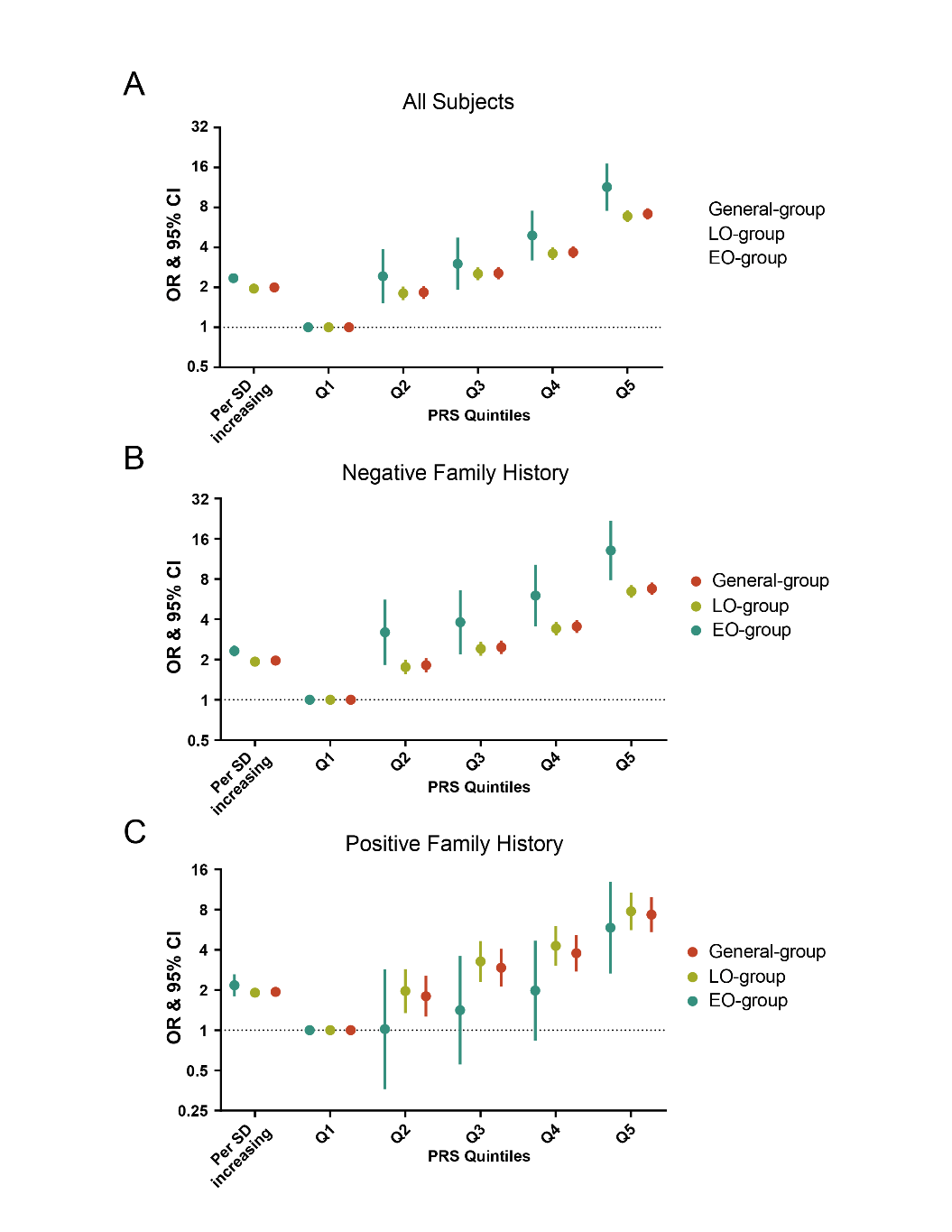


**Figure S7.** Forest plot of the heterogeneity analyses between the EOPC and LOPC risk measured by logistic regression models associated with a 269-PRS stratified by family history. PRS, polygenic risk score; EOPC, early-onset prostate cancer; LOPC, late-onset prostate cancer.


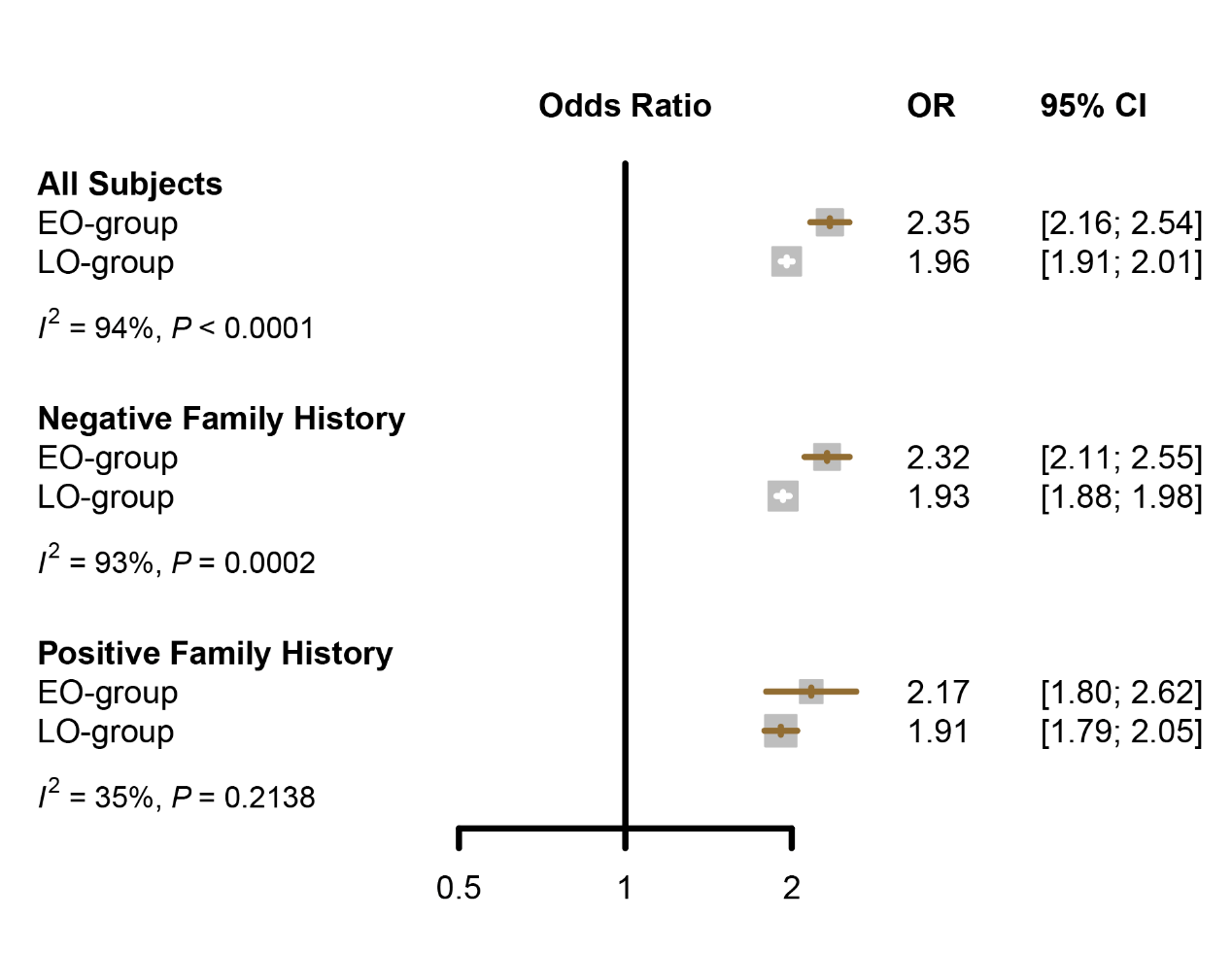


**Figure S8.** Genome-wide association studies (GWAS) for PCa risk in General-population, EO-population and LO-population using Cox proportional hazard models adjusted for age at assessment, BMI, smoking status, drinking status, assessment center and top 10 principal components, including the Manhattan plots for the General-population (A), EO-population (C) and LO-population (E), and the Q-Q plots of the General-population (B), EO-population (D) and LO-population (F). EO, early-onset; LO, late-onset.

**
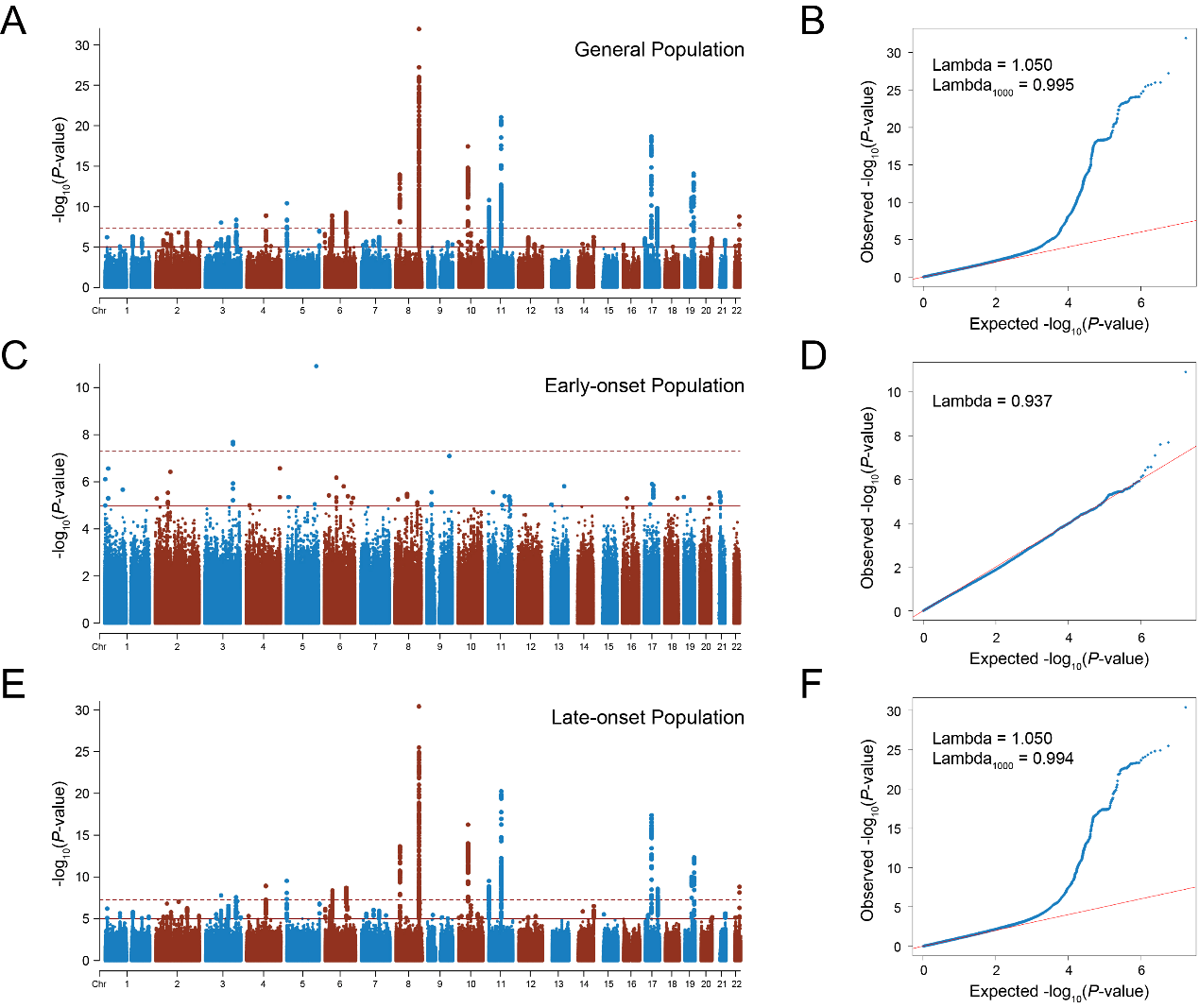
**

**Figure S9.** Forest plot of the heterogeneity analyses between the EOPC and LOPC risk associated with a General-PRS (A), an EOPC-PRS (B) and an LOPC-PRS (C) stratified by family history. PRS, polygenic risk score; EOPC, early-onset prostate cancer; LOPC, late-onset prostate cancer.


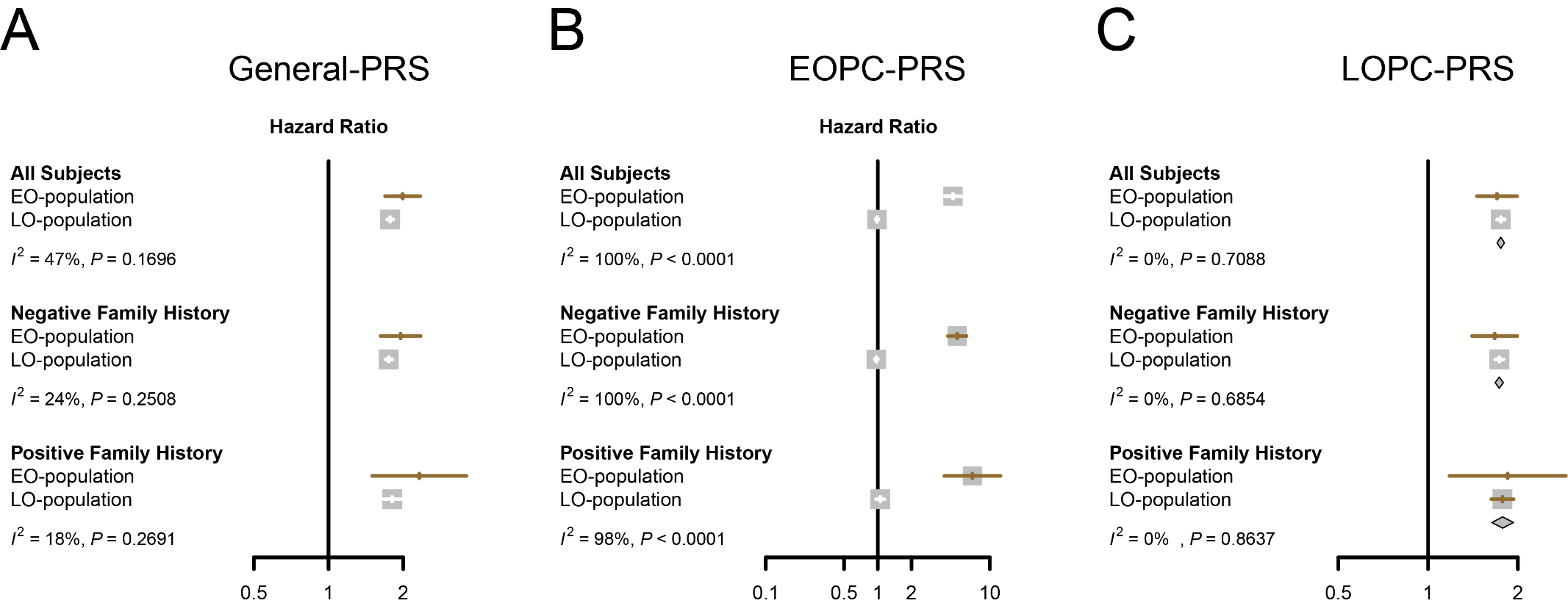


**Figure S10.** Risk estimates for PCa associated with a General-PRS (A-C), an EOPC-PRS (D-F) and an LOPC-PRS (G-I) in the General-, EO- and LO-group from the case-control population stratified by family history using logistic regression models. Models were adjusted for reference-age, BMI, smoking status, drinking status, assessment center and top 10 principal components. General-group covered all participants; EO-group was consisted of individuals with reference-age ≤ 55 years old; LO-group comprised individuals with reference-age > 55 years old. PRS, polygenic risk score; EOPC, early-onset prostate cancer; LOPC, late-onset prostate cancer; OR, odds ratio; CI, confidence interval.

**
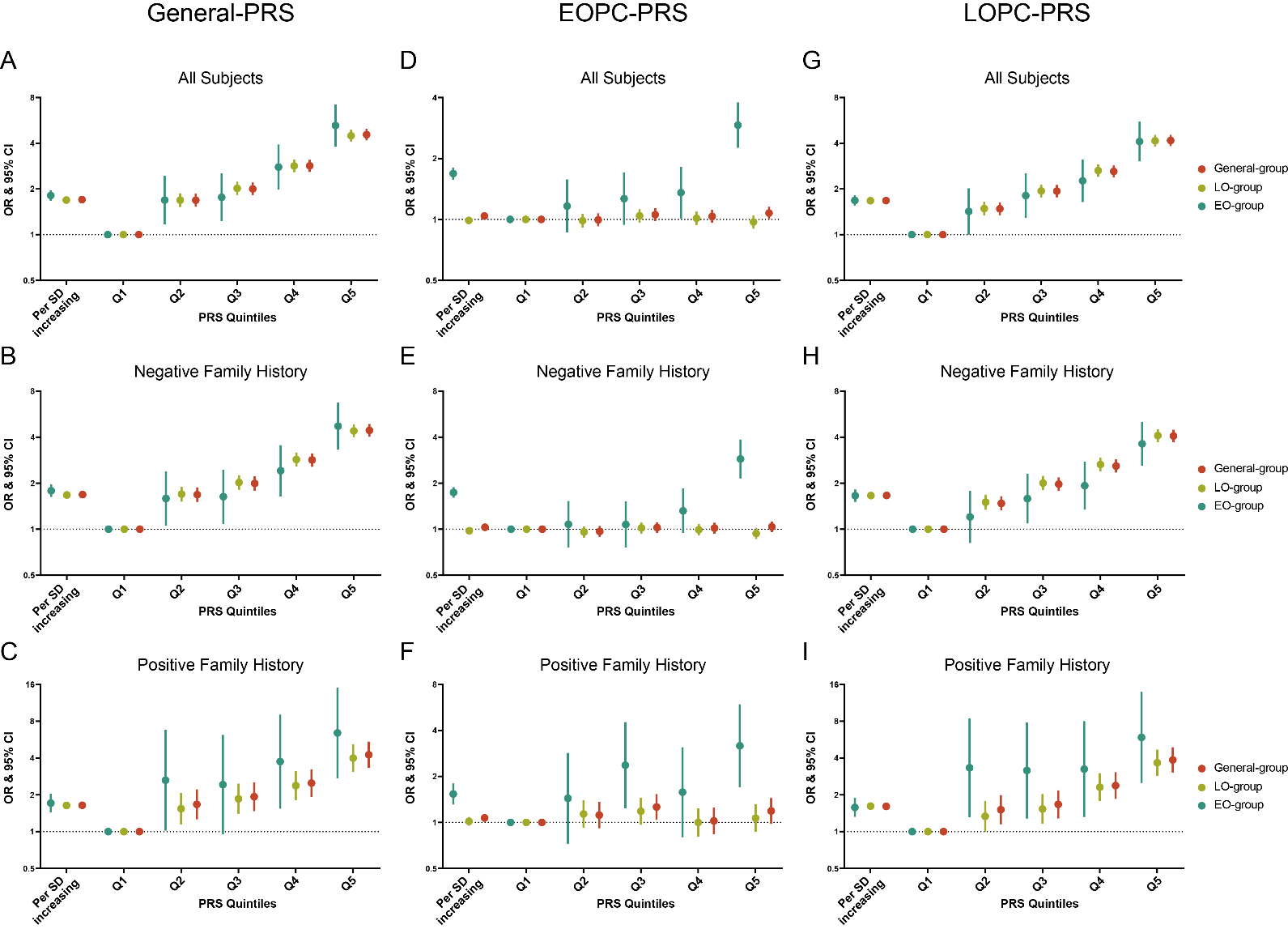
**

**Figure S11.** Forest plot of the heterogeneity analyses between the EOPC and LOPC risk measured by logistic regression models associated with a General-PRS (A), an EOPC-PRS (B) and an LOPC-PRS (C) stratified by family history. PRS, polygenic risk score; EOPC, early-onset prostate cancer; LOPC, late-onset prostate cancer.


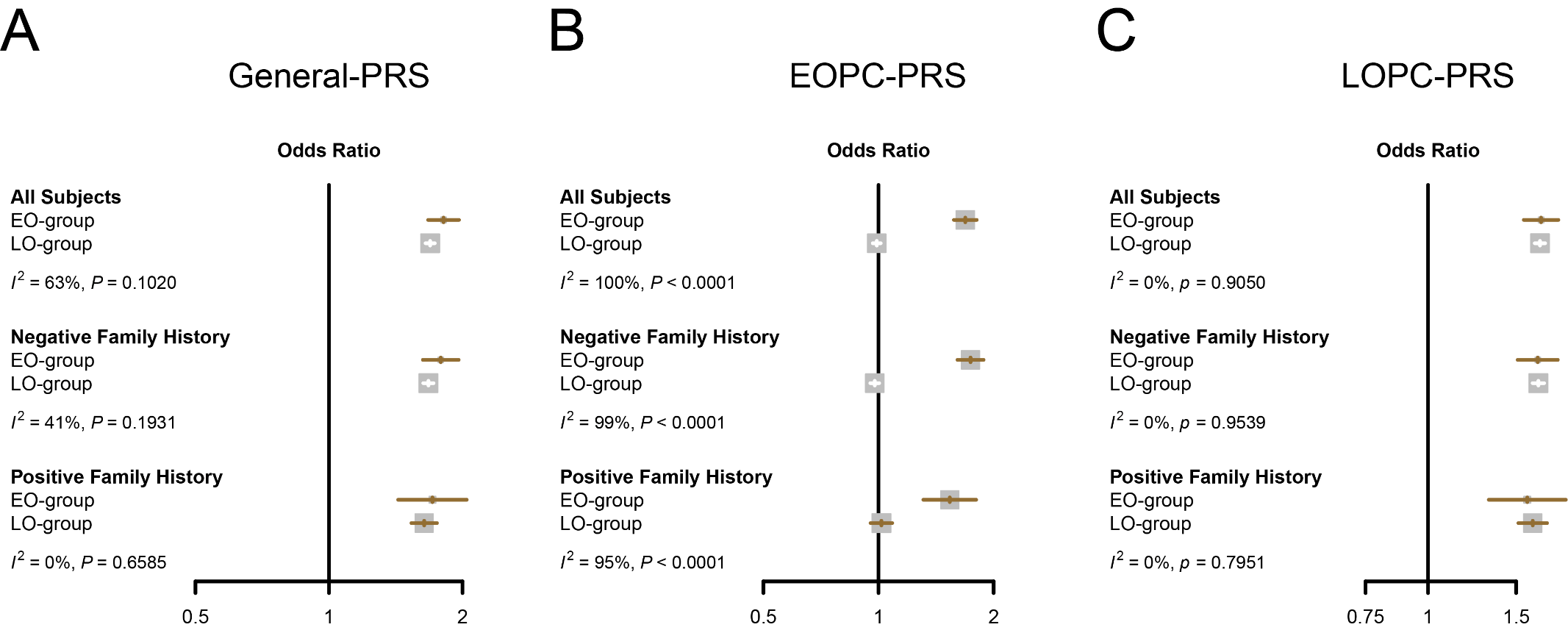


**Figure S12.** Flowchart of merging reported variants and GWAS top variants. PCa, prostate cancer; EOPC, early-onset prostate cancer; LOPC, late-onset prostate cancer.


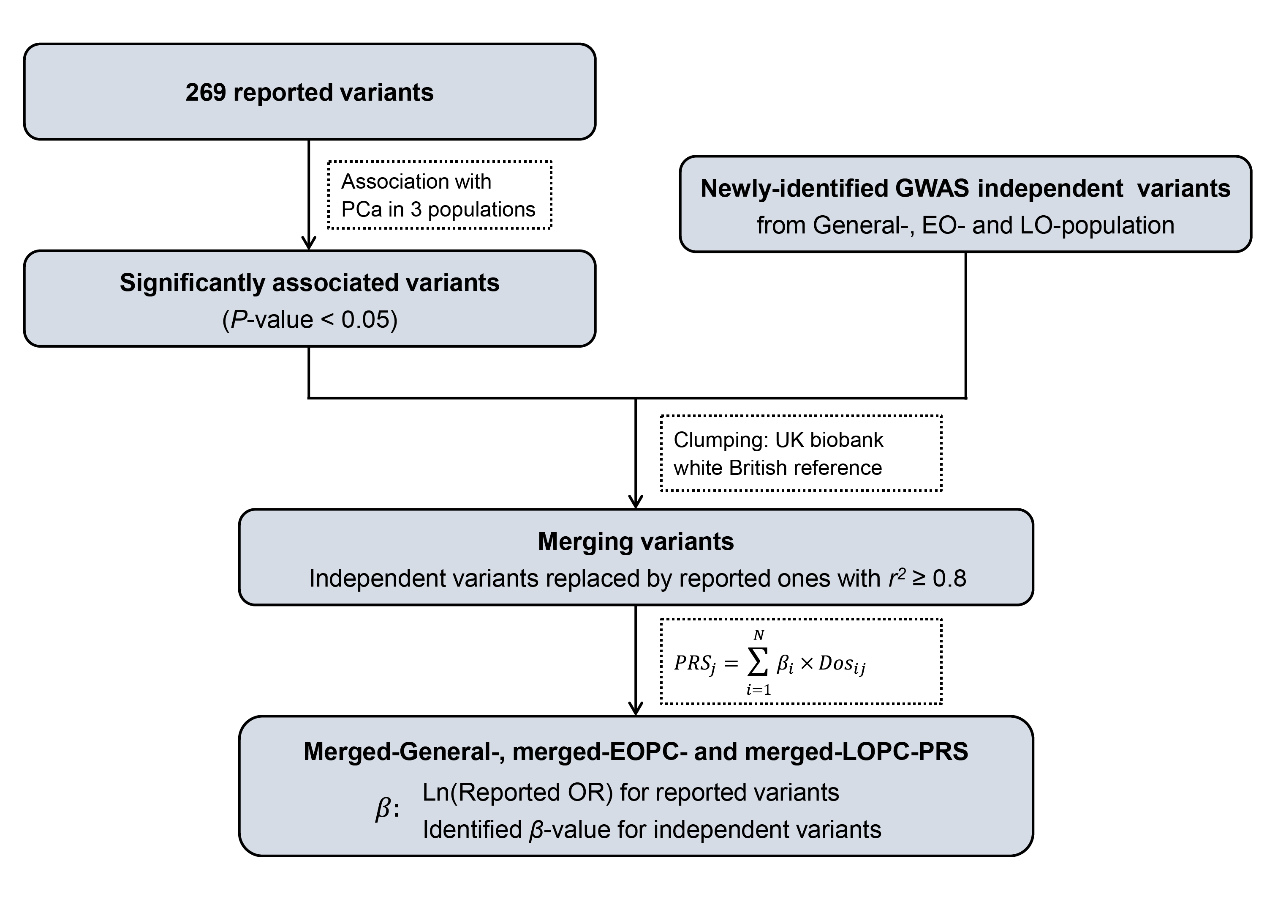


**Figure S13.** Forest plot of the heterogeneity analyses between the EOPC and LOPC risk measured by weighted Cox proportional hazard models associated with a merged-General-PRS (A), a merged-EOPC-PRS (B) and a merged-LOPC-PRS (C) stratified by family history. PRS, polygenic risk score; EOPC, early-onset prostate cancer; LOPC, late-onset prostate cancer.


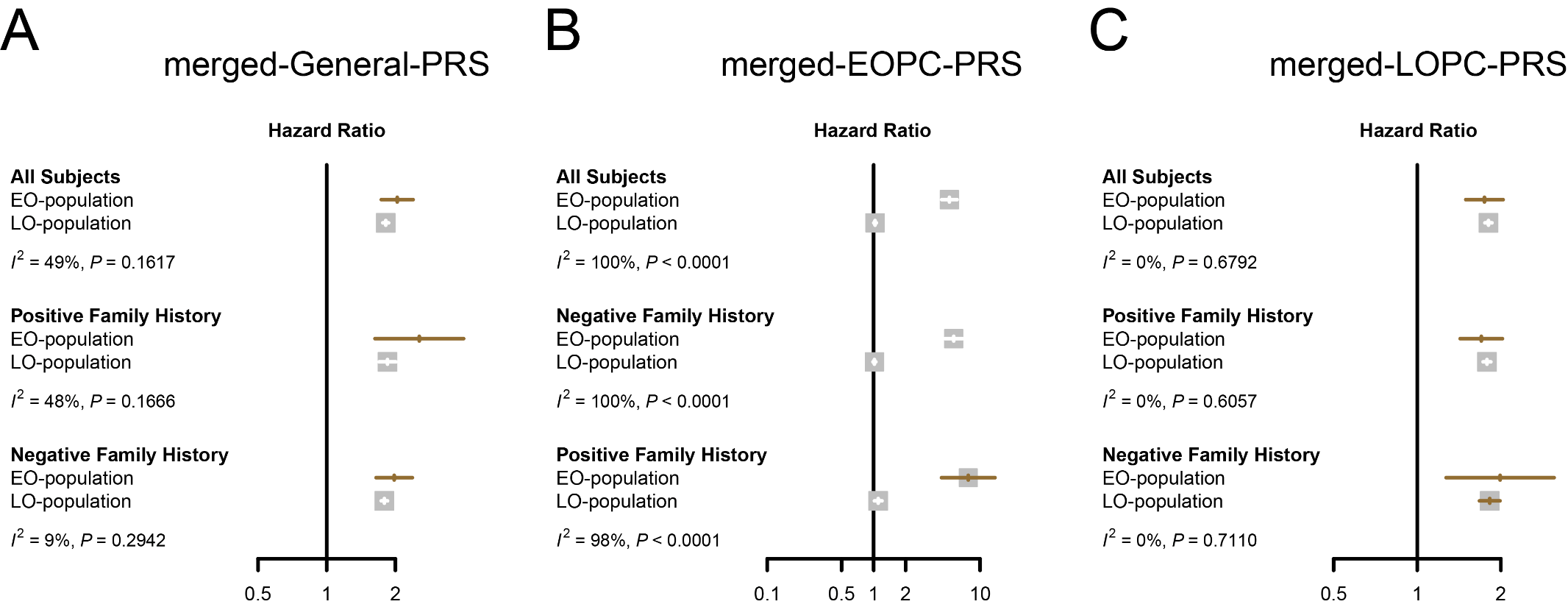


**Figure S14.** Risk estimates for PCa associated with a merged General-PRS (A-C), a merged-EOPC-PRS (D-F) and a merged-LOPC-PRS (G-I) in the General-, EO- and LO-group from the case-control population stratified by family history using logistic regression models. Models were adjusted for reference-age, BMI, smoking status, drinking status, assessment center and top 10 principal components. General-group covered all participants; EO-group was consisted of individuals with reference-age ≤ 55 years old; LO-group comprised individuals with reference-age > 55 years old. PRS, polygenic risk score; EOPC, early-onset prostate cancer; LOPC, late-onset prostate cancer; OR, odds ratio; CI, confidence interval.


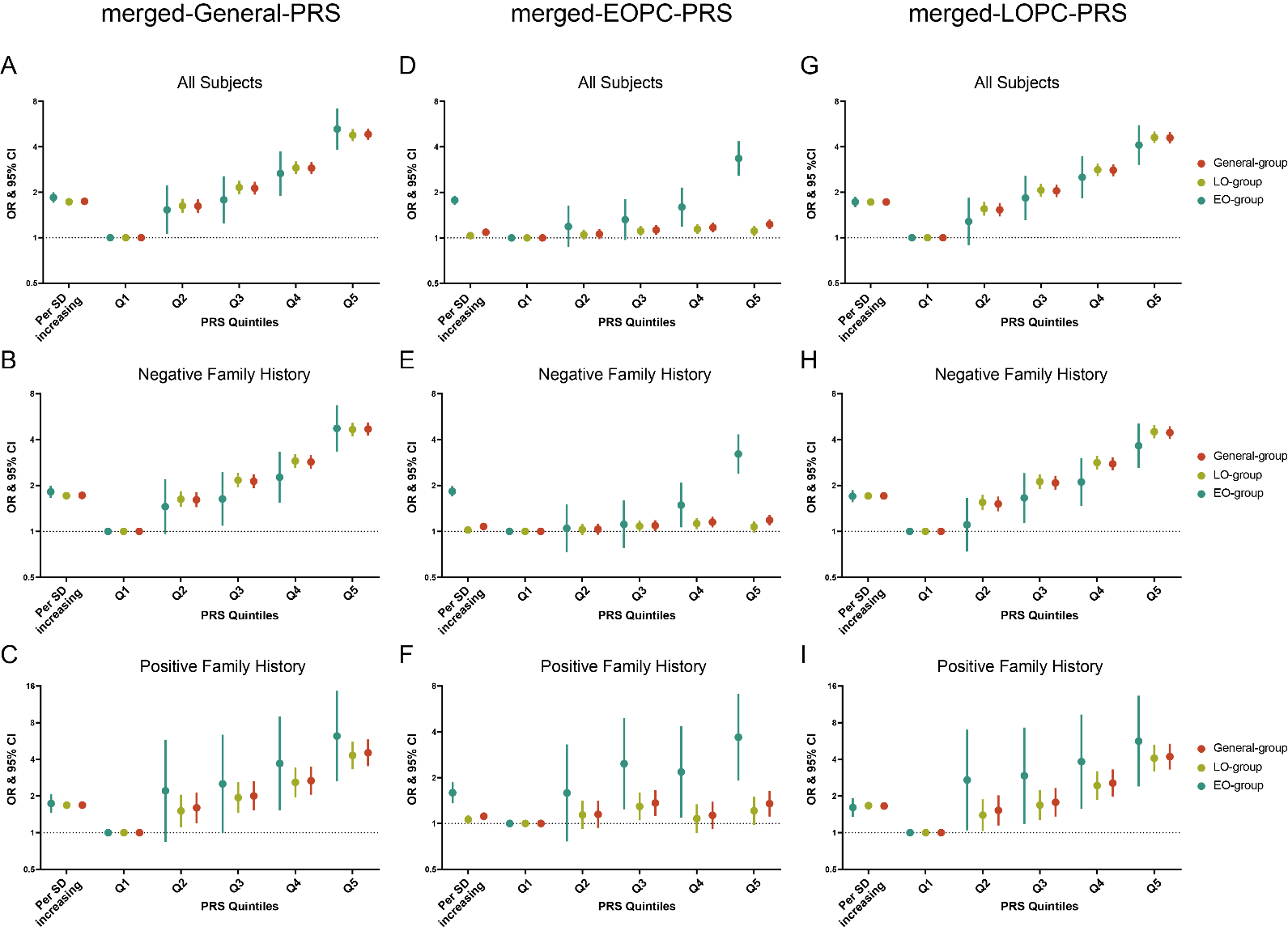


**Figure S15.** Forest plot of the heterogeneity analyses between the EOPC and LOPC risk measured by logistic regression models associated with a merged-General-PRS (A), a merged-EOPC-PRS (B) and a merged-LOPC-PRS (C) stratified by family history. PRS, polygenic risk score; EOPC, early-onset prostate cancer; LOPC, late-onset prostate cancer.


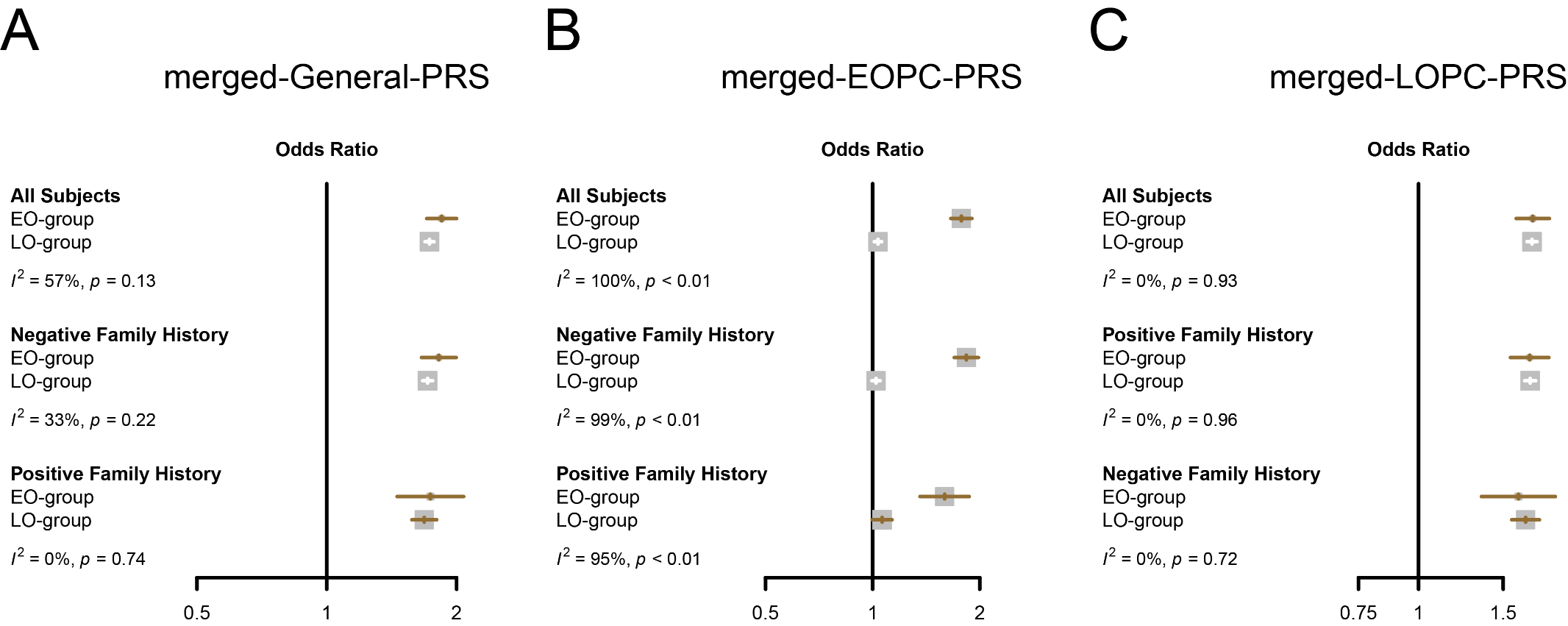


**Figure S16.** Population structure demonstrated by principal component analysis based on all high-quality SNPs. Population included participants from PLCO, TCGA and 1000 Genome Projects. PC1: Principal component 1; PC2: Principal component 2.


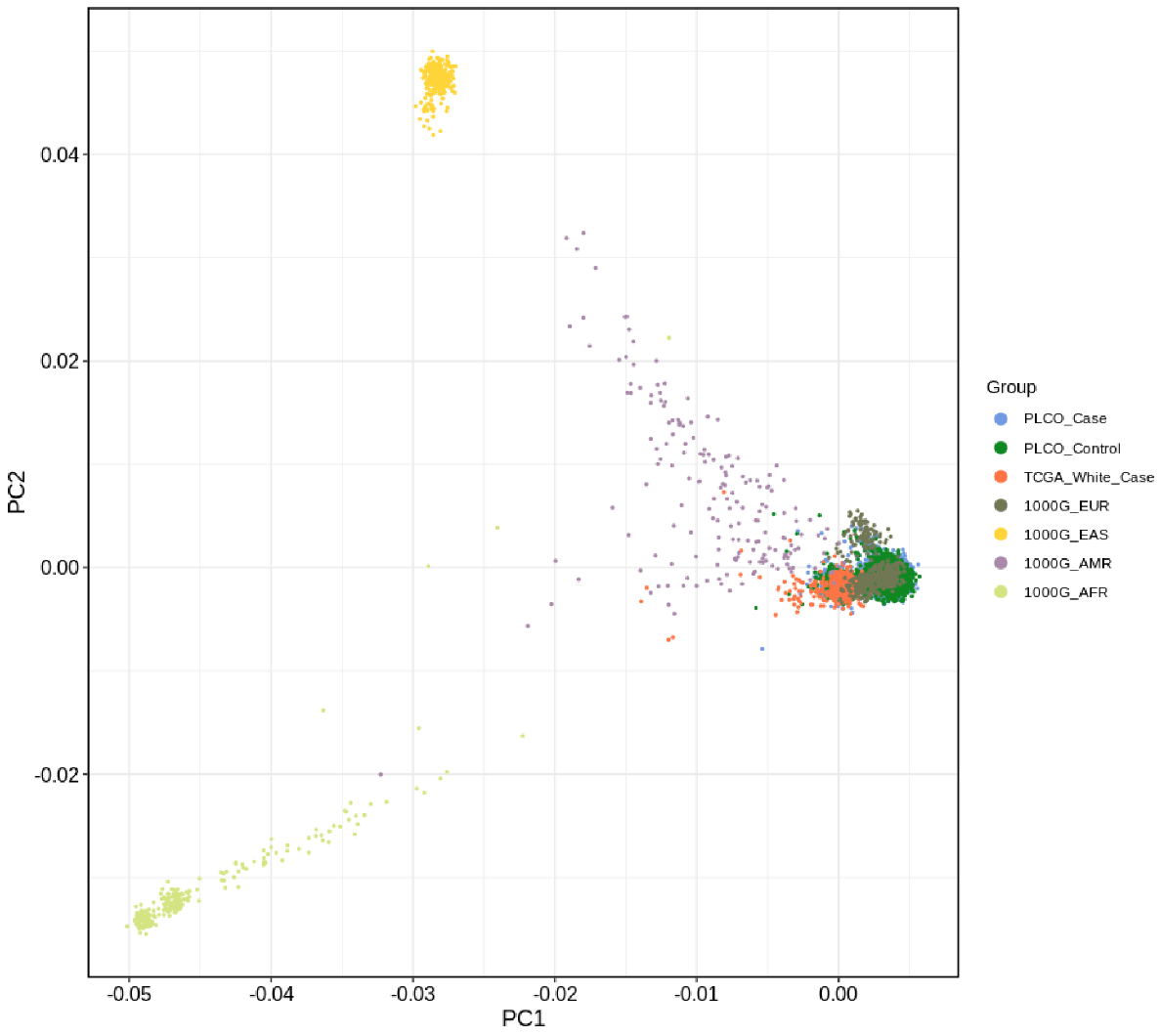


**Figure S17.** The area under the receiver operating characteristic (ROC) curve (AUC) evaluating the predictive accuracy of EOPC-PRS (**A**), 54-PRS (**B**) and 110-PRS (**C**) for EOPC in a European ancestry population generated from the PLCO cohort and TCGA program.

**
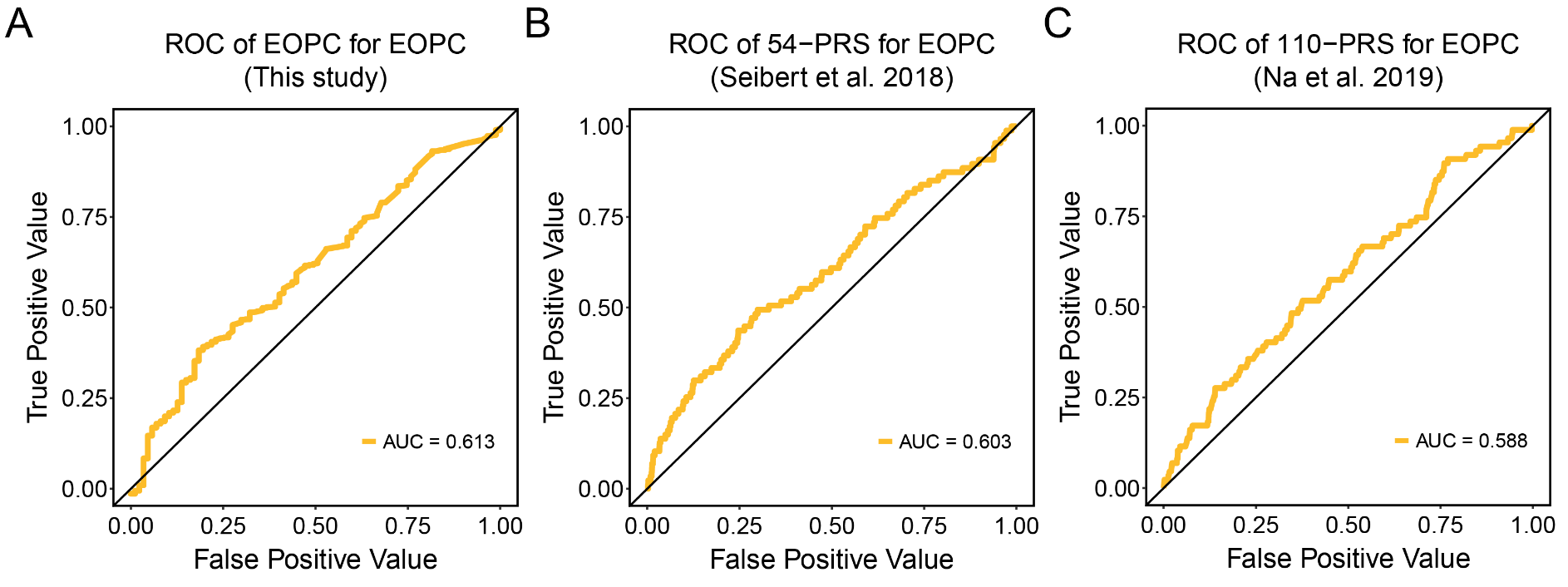
**

**Figure S18.** Time-dependent receiver operating characteristic (ROC) curves and area under the curves (AUC) from censored diagnosis data at 60-year of PSA and PSA + EOPC-PRS for prediction of PCa.

**
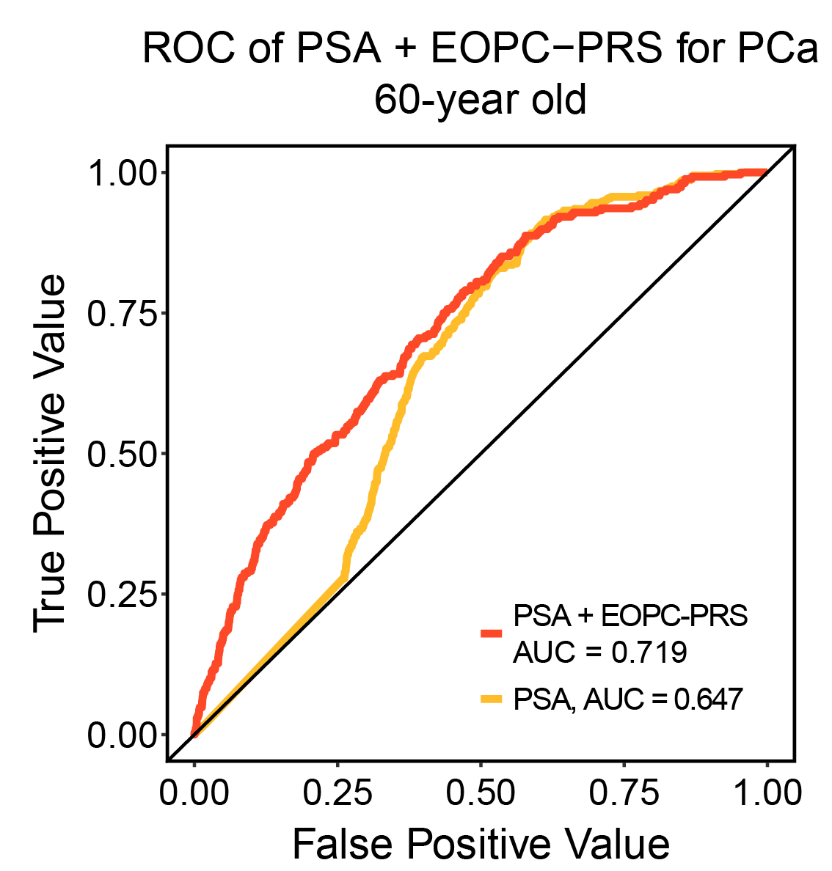
**

**Figure S19.** Risk estimates for early-onset prostate cancer (EOPC) associated with EOPC-PRS stratified by clinical variables (Gleason score, T stage and M stage).


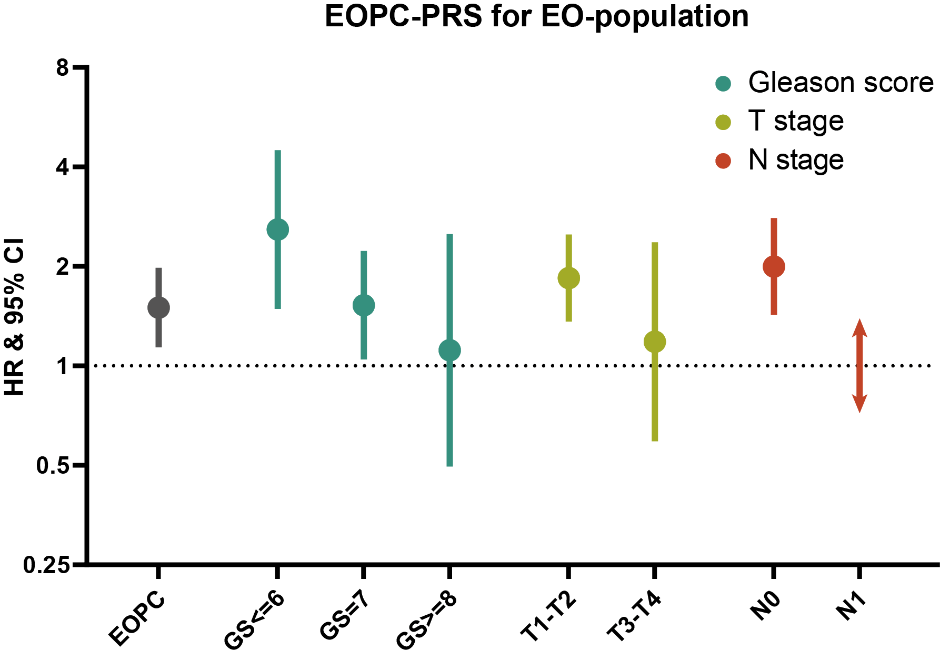


**Figure S20.** Flowchart of two-sample Mendelian randomization analyses.


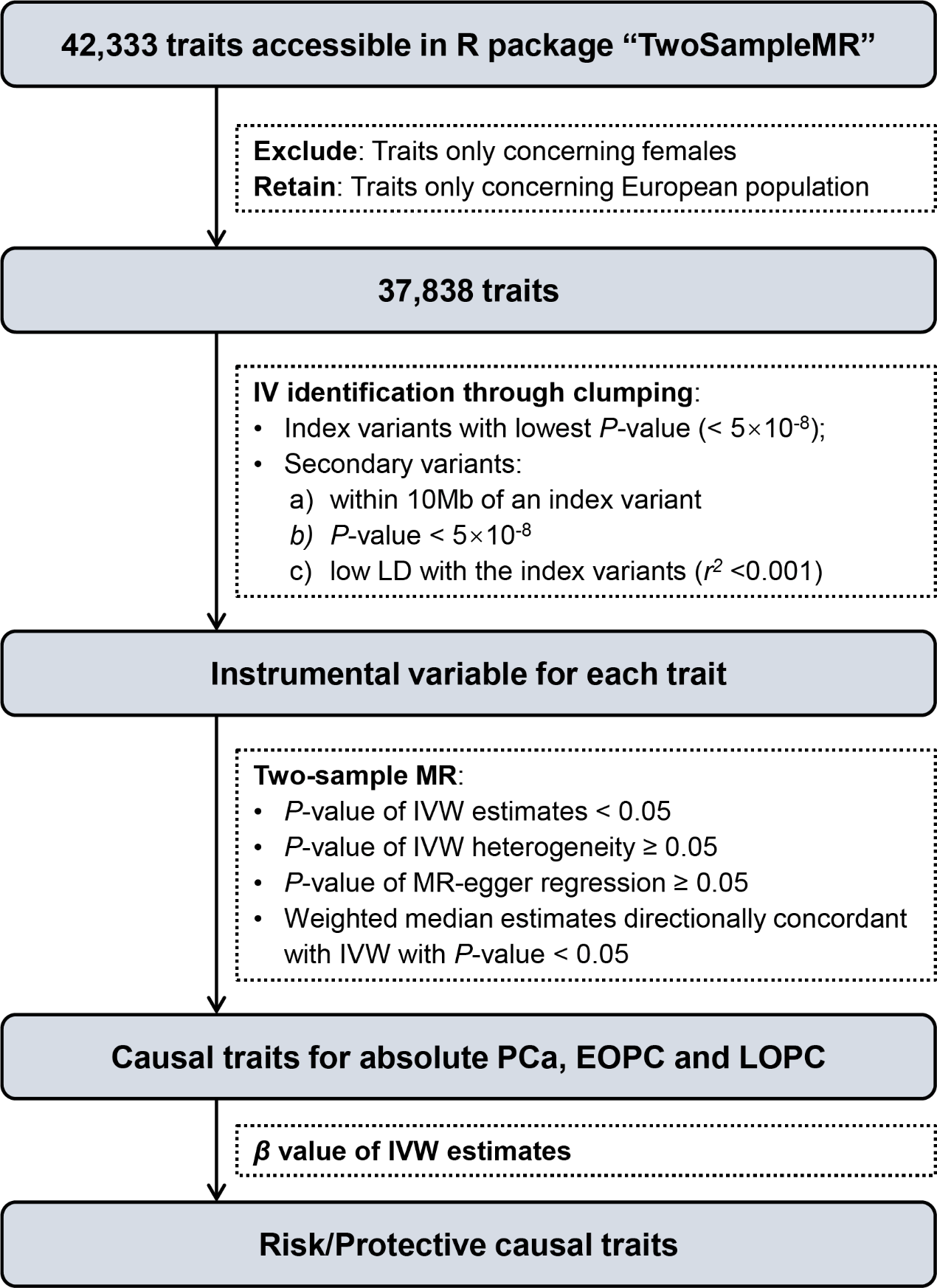


**Figure S21.** An example for the use of ProAP (Prostate cancer Age-based PheWAS). Use “Choose an outcome” and “Search” boxes to focus on the Mendelian randomization result of interest.

**
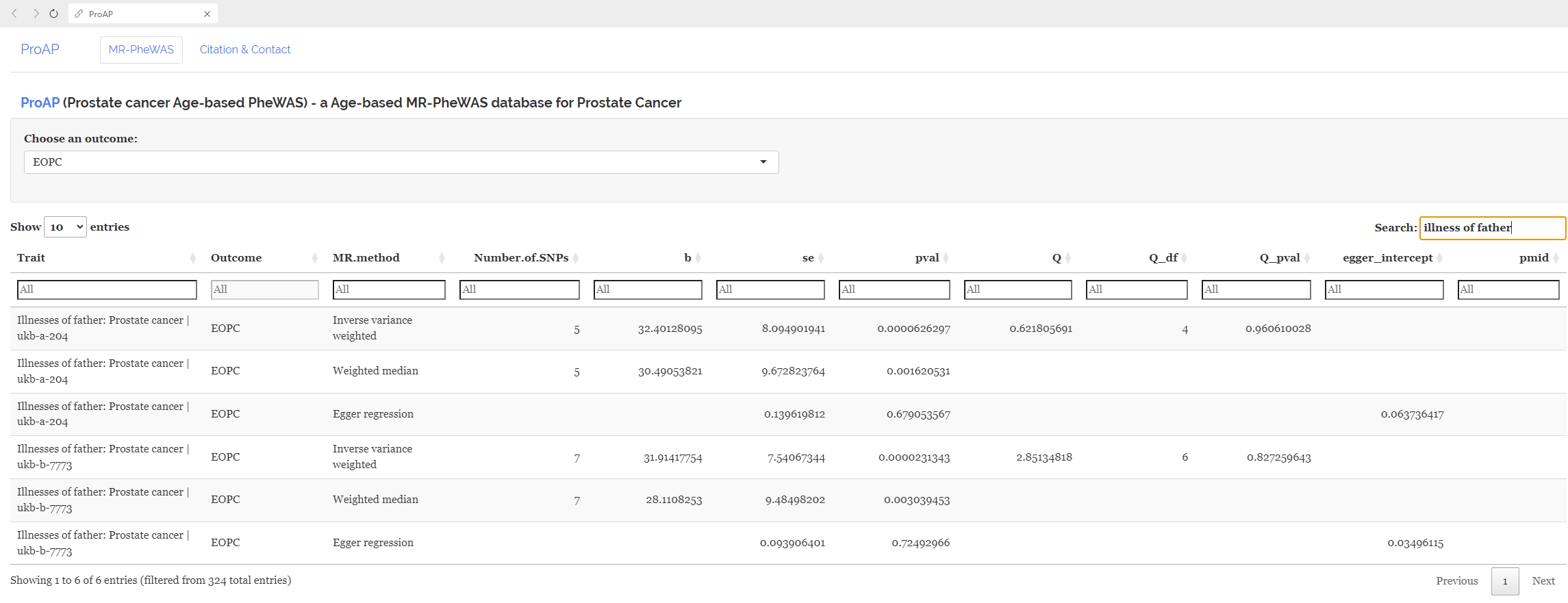
**
